# Supplementary material for: Gene and metabolite expression dependence on body mass index in human myocardium
Source: Sci Rep. 2022 Jan 26;12:1425. doi: 10.1038/s41598-022-05562-8 (PMC8791972; doi:10.1038/s41598-022-05562-8)
Supplement: Supplementary file 1 — Supplementary Information. [file 41598_2022_5562_MOESM1_ESM.pdf]

SUPPLEMENTAL MATERIAL

Gene and metabolite expression dependence on body mass index in human myocardium.

Adewale S Adebayo, Marius Roman, Syabira Yusoff, Melanie Gulston, Lathishia Joel-David, Bony Anthony, Florence Y Lai, Antonio Murgia, Bryony Eagle-Hemming, Sophia Sheikh, Tracy Kumar, Hardeep Aujla, Will Dott, Julian L Griffin, Gavin J Murphy, Marcin J Woźniak

Contents

SUPPLEMENTAL MATERIAL 1

Contents 1

Figure S1 – Plots of transcripts with the membership in significant pathways and showing biphasic BMI relationship. 2

Figure S2 – Plots of metabolite values as a function of BMI. 3

Table S1 – Breakdown of samples with transcriptomics and metabolomics analyses. 4

Table S2 – Results of the Two Lines analysis 5

Table S3 - Pre- and Post-operative characteristics in samples with transcriptomics and metabolomics analyses. 22

Table S4 – Details of the pathway analysis. 24

Table S5 – Details of genes that were highly variable between the BMI groups 27

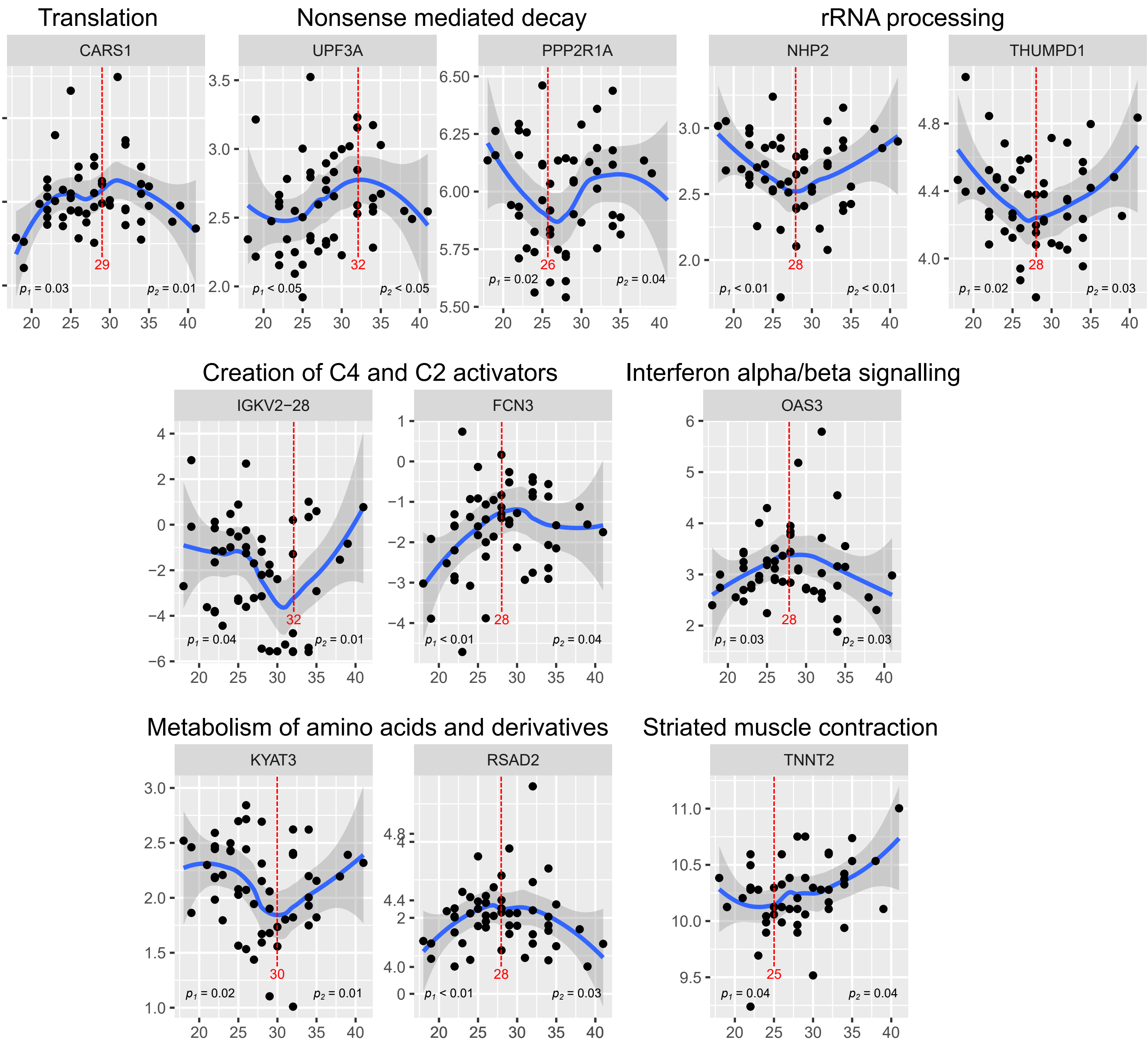

Figure S1 – Plots of transcripts with the membership in significant pathways and showing biphasic BMI relationship. The blue line shows loess regression trend. Red line indicates a breakpoint identified by the Two Line method; p1 and p2 -values indicate significant regression before or after the breakpoint.

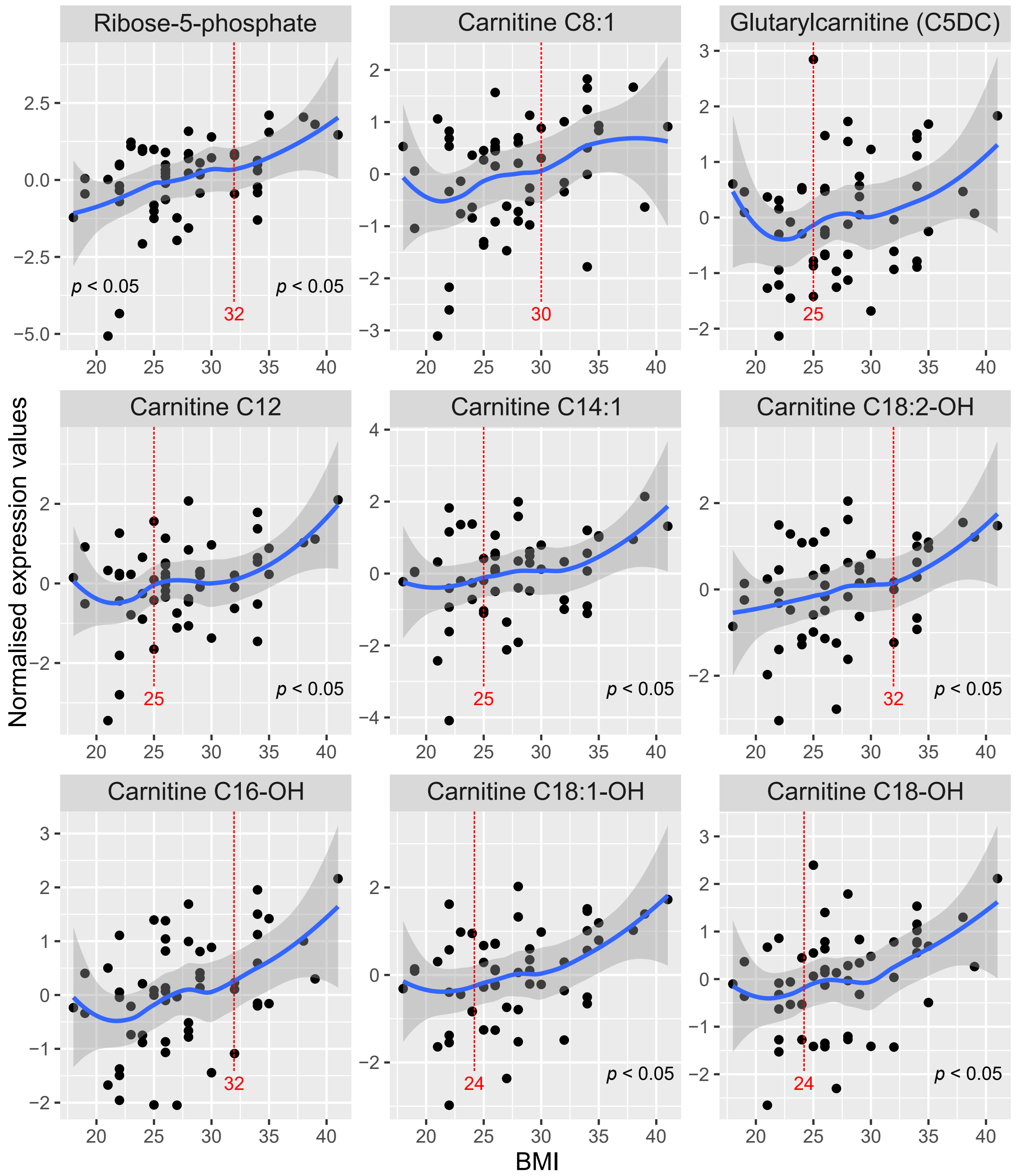

Figure S2 – Plots of metabolite normalized expression values as a function of BMI. The blue line shows loess regression trend. Red line indicates a breakpoint identified by the Two Line method; p1 and p2 -values indicate significant regression before or after the breakpoint.

Table S1 – Breakdown of samples with transcriptomics and metabolomics analyses.

ID	Transcriptomics	Metabolomics	BMI	Group
1	Yes	Yes	18	BMI<25
2	Yes	Yes	19	BMI<25
3	Yes	Yes	19	BMI<25
4	Yes	Yes	21	BMI<25
58	RIN<8	Yes	21	BMI<25
5	Yes	Yes	22	BMI<25
6	Yes	Yes	22	BMI<25
7	Yes	Yes	22	BMI<25
34	Yes	Yes	22	BMI<25
38	Yes	Yes	22	BMI<25
42	Yes	Yes	22	BMI<25
47	Yes	Yes	23	BMI<25
48	Yes	Yes	23	BMI<25
8	Yes	Yes	24	BMI<25
9	Yes	Yes	24	BMI<25
29	Yes	Yes	24	BMI<25
10	Yes	Yes	25	25≤BMI≤32
11	Yes	Yes	25	25≤BMI≤32
12	Yes	Yes	25	25≤BMI≤32
32	Yes	Yes	25	25≤BMI≤32
13	Yes	Yes	26	25≤BMI≤32
14	Yes	Yes	26	25≤BMI≤32
28	Yes	Yes	26	25≤BMI≤32
30	Yes	Yes	26	25≤BMI≤32
33	Yes	Yes	26	25≤BMI≤32
54	RIN<8	Yes	26	25≤BMI≤32
55	RIN<8	Yes	26	25≤BMI≤32
56	RIN<8	Yes	26	25≤BMI≤32
15	Yes	Yes	27	25≤BMI≤32
27	Yes	Yes	27	25≤BMI≤32
16	Yes	Yes	28	25≤BMI≤32
17	Yes	Yes	28	25≤BMI≤32
18	Yes	Yes	28	25≤BMI≤32
36	Yes	Yes	28	25≤BMI≤32
41	Yes	Yes	28	25≤BMI≤32
19	Yes	Yes	29	25≤BMI≤32
20	Yes	Yes	29	25≤BMI≤32
35	Yes	Yes	29	25≤BMI≤32
49	Yes	Yes	29	25≤BMI≤32
21	Yes	Yes	30	25≤BMI≤32
45	Yes	Yes	30	25≤BMI≤32
53	Yes	NA	31	25≤BMI≤32
22	Yes	Yes	32	25≤BMI≤32
43	Yes	removed	32	25≤BMI≤32
25	Yes	Yes	32	25≤BMI≤32
40	Yes	Yes	32	25≤BMI≤32
52	Yes	NA	32	25≤BMI≤32
26	Yes	Yes	34	BMI>32
39	Yes	Yes	34	BMI>32
44	Yes	Yes	34	BMI>32
57	RIN<8	Yes	34	BMI>32
46	Yes	Yes	34	BMI>32
50	Yes	Yes	34	BMI>32
23	Yes	Yes	35	BMI>32
31	Yes	Yes	35	BMI>32
37	Yes	Yes	38	BMI>32
51	Yes	Yes	39	BMI>32
24	Yes	Yes	41	BMI>32
59	NA	NA	29	25≤BMI≤32
60	NA	NA	27	25≤BMI≤32
61	NA	NA	33	BMI>32
62	NA	NA	29	25≤BMI≤32
63	NA	NA	29	25≤BMI≤32
64	NA	NA	28	25≤BMI≤32
65	NA	NA	32	25≤BMI≤32
66	NA	NA	28	25≤BMI≤32

Table S2 – Results of the Two Lines analysis for all transcripts significant for both regression lines and for metabolites significant between the analyzed group. z1, z2 – test statistics for both regression lines; b1, b2 – regression coefficients, p1, p2 – p-values for each regression line.

Ensembl ID	Gene description	Gene/metabolite name	Type	b1	z1	p1	b2	z2	p2	breakpoint
ENSG00000272768	novel transcript, antisense to PURB	AC004854.2	lncRNA	-0.446	-3.137	0.002	0.090	2.637	0.008	24
ENSG00000269696	novel transcript, antisense to ZFP28	AC005498.3	lncRNA	-0.209	-2.230	0.026	0.081	2.182	0.029	25
ENSG00000224063	novel transcript, antisense to TFPI and CALCRL	AC007319.1	lncRNA	0.163	2.556	0.011	-0.113	-2.839	0.005	28
ENSG00000279249	novel transcript, antisense to CBLN1	AC007614.1	lncRNA	0.189	2.883	0.004	-0.180	-2.176	0.030	28
ENSG00000267179	novel protein	AC008770.2	protein_coding	-0.184	-2.105	0.035	0.257	3.266	0.001	29
ENSG00000279598	novel transcript, antisense to titin	AC009948.3	TEC	0.131	2.658	0.008	-0.121	-2.618	0.009	26
ENSG00000272622	novel transcript	AC010735.2	lncRNA	-0.138	-2.170	0.030	0.042	2.067	0.039	26
ENSG00000285596	novel transcript	AC017116.2	lncRNA	0.151	2.428	0.015	-0.116	-2.319	0.020	28
ENSG00000186019	novel transcript, antisense to ZNF225 and ZNF224	AC021092.1	lncRNA	0.096	2.072	0.038	-0.089	-2.863	0.004	30
ENSG00000248115	novel transcript	AC023154.1	lncRNA	-0.083	-2.171	0.030	0.059	2.625	0.009	28
ENSG00000273771	novel transcript	AC024337.2	lncRNA	-0.102	-2.679	0.007	0.087	3.059	0.002	27
ENSG00000273329	novel transcript	AC078846.1	lncRNA	0.038	2.365	0.018	-0.112	-2.986	0.003	32
ENSG00000253190	novel transcript	AC084082.1	lncRNA	-0.183	-2.264	0.024	0.113	3.199	0.001	26
ENSG00000267127	novel protein	AC090360.1	protein_coding	0.100	2.769	0.006	-0.186	-3.917	0.000	32
ENSG00000279283	novel transcript	AC131009.4	TEC	0.115	2.272	0.023	-0.096	-2.424	0.015	28
ENSG00000279204	TEC	AC134043.3	TEC	-0.167	-2.674	0.008	0.065	2.122	0.034	26
ENSG00000182827	acyl-CoA binding domain containing 3 [Source:HGNC Symbol;Acc:HGNC:15453]	ACBD3	protein_coding	-0.041	-2.533	0.011	0.018	2.151	0.032	26
ENSG00000118507	A-kinase anchoring protein 7 [Source:HGNC Symbol;Acc:HGNC:377]	AKAP7	protein_coding	-0.076	-2.601	0.009	0.083	3.676	0.000	28

ENSG00000285723	novel protein	AL034430.2	protein_coding	0.070	1.997	0.046	-0.073	-2.264	0.024	29
ENSG00000276809	novel transcript, sense intronic to DNAJC3	AL138955.1	lncRNA	-0.279	-4.095	0.000	0.088	2.432	0.015	26
ENSG00000187186	uncharacterized LOC730098 [Source:NCBI gene (formerly Entrezgene);Acc:730098]	AL162231.1	protein_coding	-0.108	-1.983	0.047	0.078	1.972	0.049	26
ENSG00000269896	small nuclear ribonucleoprotein N (SNRPN) pseudogene	AL513477.1	transcribed_processed_pseudogene	0.095	2.243	0.025	-0.154	-2.569	0.010	28
ENSG00000253710	ALG11 alpha-1,2-mannosyltransferase [Source:HGNC Symbol;Acc:HGNC:32456]	ALG11	protein_coding	0.034	2.115	0.034	-0.025	-2.198	0.028	29
ENSG00000139645	ankyrin repeat domain 52 [Source:HGNC Symbol;Acc:HGNC:26614]	ANKRD52	protein_coding	0.047	2.300	0.021	-0.045	-2.323	0.020	28
ENSG00000151572	anoctamin 4 [Source:HGNC Symbol;Acc:HGNC:23837]	ANO4	protein_coding	0.100	2.381	0.017	-0.198	-3.228	0.001	29
ENSG00000106367	adaptor related protein complex 1 subunit sigma 1 [Source:HGNC Symbol;Acc:HGNC:559]	AP1S1	protein_coding	-0.040	-2.176	0.030	0.048	2.137	0.033	30
ENSG00000136044	adaptor protein, phosphotyrosine interacting with PH domain and leucine zipper 2 [Source:HGNC Symbol;Acc:HGNC:18242]	APPL2	protein_coding	0.053	2.519	0.012	-0.040	-2.633	0.008	28
ENSG00000177479	ariadne RBR E3 ubiquitin protein ligase 2 [Source:HGNC Symbol;Acc:HGNC:690]	ARIH2	protein_coding	-0.072	-2.156	0.031	0.011	1.983	0.047	24
ENSG00000177917	ADP ribosylation factor like GTPase 6 interacting protein 6 [Source:HGNC Symbol;Acc:HGNC:24048]	ARL6IP6	protein_coding	-0.106	-2.635	0.008	0.054	2.031	0.042	27
ENSG00000224470	ataxin 1 like [Source:HGNC Symbol;Acc:HGNC:33279]	ATXN1L	protein_coding	-0.024	-3.174	0.002	0.021	2.745	0.006	28
ENSG00000105778	AVL9 cell migration associated [Source:HGNC Symbol;Acc:HGNC:28994]	AVL9	protein_coding	0.048	2.834	0.005	-0.024	-1.971	0.049	26
ENSG00000162630	beta-1,3-galactosyltransferase 2 [Source:HGNC Symbol;Acc:HGNC:917]	B3GALT2	protein_coding	-0.110	-2.674	0.007	0.038	2.138	0.032	25
ENSG00000123810	B9 domain containing 2 [Source:HGNC	B9D2	protein_coding	0.143	2.186	0.029	-0.082	-2.050	0.040	28

	Symbol;Acc:HGNC:28636]									
ENSG00000169925	bromodomain containing 3 [Source:HGNC Symbol;Acc:HGNC:1104]	BRD3	protein_coding	0.037	2.912	0.004	-0.039	-2.805	0.005	29
ENSG00000164061	bassoon presynaptic cytomatrix protein [Source:HGNC Symbol;Acc:HGNC:1117]	BSN	protein_coding	0.219	3.092	0.002	-0.066	-2.848	0.004	25
ENSG00000148925	BTB domain containing 10 [Source:HGNC Symbol;Acc:HGNC:21445]	BTBD10	protein_coding	-0.057	-3.007	0.003	0.024	2.199	0.028	27
ENSG00000240809	CAP1 pseudogene 1 [Source:HGNC Symbol;Acc:HGNC:31134]	CAP1P1	processed_pseudogene	-0.084	-2.217	0.027	0.090	2.214	0.027	29
ENSG00000110619	cysteinyl-tRNA synthetase 1 [Source:HGNC Symbol;Acc:HGNC:1493]	CARS1	protein_coding	0.041	2.210	0.027	-0.044	-2.616	0.009	29
ENSG00000165813	coiled-coil domain containing 186 [Source:HGNC Symbol;Acc:HGNC:24349]	CCDC186	protein_coding	-0.037	-2.090	0.037	0.023	2.444	0.015	25
ENSG00000166510	coiled-coil domain containing 68 [Source:HGNC Symbol;Acc:HGNC:24350]	CCDC68	protein_coding	0.066	2.633	0.008	-0.063	-3.113	0.002	28
ENSG00000275385	C-C motif chemokine ligand 18 [Source:HGNC Symbol;Acc:HGNC:10616]	CCL18	protein_coding	-0.186	-2.677	0.007	0.414	3.641	0.000	32
ENSG00000149654	cadherin 22 [Source:HGNC Symbol;Acc:HGNC:13251]	CDH22	protein_coding	0.111	2.179	0.029	-0.162	-2.528	0.011	28
ENSG00000213753	CENPB DNA-binding domains containing 1 pseudogene 1 [Source:HGNC Symbol;Acc:HGNC:28421]	CENPBD1P1	transcribed_processed_pseudogene	-0.069	-2.235	0.025	0.078	3.088	0.002	27
ENSG00000172831	carboxylesterase 2 [Source:HGNC Symbol;Acc:HGNC:1864]	CES2	protein_coding	0.027	2.195	0.028	-0.024	-2.540	0.011	28
ENSG00000116785	complement factor H related 3 [Source:HGNC Symbol;Acc:HGNC:16980]	CFHR3	protein_coding	-0.343	-2.763	0.006	0.106	2.179	0.029	25
ENSG00000125611	coiled-coil-helix-coiled-coil-helix domain containing 5 [Source:HGNC Symbol;Acc:HGNC:17840]	CHCHD5	protein_coding	-0.050	-2.142	0.032	0.051	2.135	0.033	29
ENSG00000090539	chordin [Source:HGNC Symbol;Acc:HGNC:1949]	CHRD	protein_coding	0.075	2.358	0.018	-0.107	-4.389	0.000	29
ENSG00000247572	CKMT2 antisense RNA 1 [Source:HGNC	CKMT2-AS1	lncRNA	0.114	2.156	0.031	-0.054	-2.442	0.015	28

	Symbol;Acc:HGNC:48997]									
ENSG00000175505	cardiotrophin like cytokine factor 1 [Source:HGNC Symbol;Acc:HGNC:17412]	CLCF1	protein_coding	-0.070	-2.651	0.008	0.114	2.587	0.010	32
ENSG00000111729	C-type lectin domain family 4 member A [Source:HGNC Symbol;Acc:HGNC:13257]	CLEC4A	protein_coding	-0.204	-2.578	0.010	0.285	3.403	0.001	30
ENSG00000198791	CCR4-NOT transcription complex subunit 7 [Source:HGNC Symbol;Acc:HGNC:14101]	CNOT7	protein_coding	-0.018	-2.191	0.028	0.018	2.200	0.028	28
ENSG00000136943	cathepsin V [Source:HGNC Symbol;Acc:HGNC:2538]	CTSV	protein_coding	-0.098	-1.962	0.050	0.248	2.919	0.004	29
ENSG00000132716	DDB1 and CUL4 associated factor 8 [Source:HGNC Symbol;Acc:HGNC:24891]	DCAF8	protein_coding	0.039	2.759	0.006	-0.033	-2.178	0.029	26
ENSG00000145214	diacylglycerol kinase theta [Source:HGNC Symbol;Acc:HGNC:2856]	DGKQ	protein_coding	0.223	3.617	0.000	-0.056	-2.940	0.003	25
ENSG00000138246	DnaJ heat shock protein family (Hsp40) member C13 [Source:HGNC Symbol;Acc:HGNC:30343]	DNAJC13	protein_coding	0.028	3.625	0.000	-0.038	-2.974	0.003	29
ENSG00000178498	deltex E3 ubiquitin ligase 3 [Source:HGNC Symbol;Acc:HGNC:24457]	DTX3	protein_coding	0.018	2.124	0.034	-0.040	-2.110	0.035	32
ENSG00000243701	DPPA2 upstream binding RNA [Source:HGNC Symbol;Acc:HGNC:48569]	DUBR	lncRNA	0.058	2.567	0.010	-0.047	-2.271	0.023	29
ENSG00000093144	ethylmalonyl-CoA decarboxylase 1 [Source:HGNC Symbol;Acc:HGNC:21489]	ECHDC1	protein_coding	0.064	3.604	0.000	-0.022	-2.117	0.034	26
ENSG00000203734	epithelial cell transforming 2 like [Source:HGNC Symbol;Acc:HGNC:21118]	ECT2L	protein_coding	-0.387	-2.423	0.015	0.094	2.022	0.043	25
ENSG00000186976	EF-hand calcium binding domain 6 [Source:HGNC Symbol;Acc:HGNC:24204]	EFCAB6	protein_coding	-0.151	-2.813	0.005	0.051	2.123	0.034	26
ENSG00000145242	EPH receptor A5 [Source:HGNC Symbol;Acc:HGNC:3389]	EPHA5	protein_coding	0.109	2.648	0.008	-0.132	-2.722	0.006	29
ENSG00000089248	endoplasmic reticulum protein 29 [Source:HGNC Symbol;Acc:HGNC:13799]	ERP29	protein_coding	0.035	2.037	0.042	-0.018	-2.599	0.009	26
ENSG00000157557	ETS proto-oncogene 2, transcription factor	ETS2	protein_coding	-0.037	-2.199	0.028	0.045	2.913	0.004	28

	[Source:HGNC Symbol;Acc:HGNC:3489]									
ENSG00000184083	family with sequence similarity 120C [Source:HGNC Symbol;Acc:HGNC:16949]	FAM120C	protein_coding	-0.073	-2.155	0.031	0.043	2.208	0.027	28
ENSG00000221909	family with sequence similarity 200 member A [Source:HGNC Symbol;Acc:HGNC:25401]	FAM200A	protein_coding	-0.104	-2.723	0.006	0.045	2.194	0.028	26
ENSG00000071859	family with sequence similarity 50 member A [Source:HGNC Symbol;Acc:HGNC:18786]	FAM50A	protein_coding	-0.066	-5.312	0.000	0.022	2.051	0.040	26
ENSG00000167196	F-box protein 22 [Source:HGNC Symbol;Acc:HGNC:13593]	FBXO22	protein_coding	-0.114	-2.683	0.007	0.029	2.077	0.038	25
ENSG00000142748	ficolin 3 [Source:HGNC Symbol;Acc:HGNC:3625]	FCN3	protein_coding	0.182	3.313	0.001	-0.067	-2.052	0.040	28
ENSG00000109158	gamma-aminobutyric acid type A receptor subunit alpha4 [Source:HGNC Symbol;Acc:HGNC:4078]	GABRA4	protein_coding	-0.391	-3.287	0.001	0.143	2.749	0.006	26
ENSG00000136542	polypeptide N-acetylgalactosaminyltransferase 5 [Source:HGNC Symbol;Acc:HGNC:4127]	GALNT5	protein_coding	0.076	2.960	0.003	-0.061	-2.015	0.044	29
ENSG00000005436	GC-rich sequence DNA-binding factor 2 [Source:HGNC Symbol;Acc:HGNC:1317]	GCFC2	protein_coding	-0.128	-4.328	0.000	0.041	2.975	0.003	25
ENSG00000162676	growth factor independent 1 transcriptional repressor [Source:HGNC Symbol;Acc:HGNC:4237]	GFI1	protein_coding	0.176	2.324	0.020	-0.163	-2.387	0.017	29
ENSG00000163655	guanine monophosphate synthase [Source:HGNC Symbol;Acc:HGNC:4378]	GMPS	protein_coding	-0.027	-2.266	0.023	0.035	2.255	0.024	29
ENSG00000087258	G protein subunit alpha o1 [Source:HGNC Symbol;Acc:HGNC:4389]	GNAO1	protein_coding	0.040	1.996	0.046	-0.033	-2.264	0.024	29
ENSG00000108433	golgi SNAP receptor complex member 2 [Source:HGNC Symbol;Acc:HGNC:4431]	GOSR2	protein_coding	-0.038	-2.022	0.043	0.050	3.793	0.000	29
ENSG00000159592	GC-rich promoter binding protein 1 like 1 [Source:HGNC Symbol;Acc:HGNC:28843]	GPBP1L1	protein_coding	-0.037	-2.382	0.017	0.013	2.529	0.011	25
ENSG00000156097	G protein-coupled receptor 61 [Source:HGNC Symbol;Acc:HGNC:13300]	GPR61	protein_coding	0.172	2.217	0.027	-0.183	-2.579	0.010	28

ENSG00000123901	G protein-coupled receptor 83 [Source:HGNC Symbol;Acc:HGNC:4523]	GPR83	protein_coding	0.137	2.406	0.016	-0.099	-1.990	0.047	28
ENSG00000125675	glutamate ionotropic receptor AMPA type subunit 3 [Source:HGNC Symbol;Acc:HGNC:4573]	GRIA3	protein_coding	0.072	2.179	0.029	-0.108	-2.497	0.013	32
ENSG00000228315	GUSB pseudogene 11 [Source:HGNC Symbol;Acc:HGNC:42325]	GUSBP11	lncRNA	0.105	2.404	0.016	-0.065	-2.843	0.004	26
ENSG00000092036	HAUS augmin like complex subunit 4 [Source:HGNC Symbol;Acc:HGNC:20163]	HAUS4	protein_coding	-0.041	-2.124	0.034	0.038	2.480	0.013	29
ENSG00000249115	HAUS augmin like complex subunit 5 [Source:HGNC Symbol;Acc:HGNC:29130]	HAUS5	protein_coding	0.112	2.836	0.005	-0.070	-2.964	0.003	28
ENSG00000162639	HEN methyltransferase 1 [Source:HGNC Symbol;Acc:HGNC:26400]	HENMT1	protein_coding	-0.083	-2.742	0.006	0.104	2.909	0.004	29
ENSG00000169660	hexosaminidase D [Source:HGNC Symbol;Acc:HGNC:26307]	HEXD	protein_coding	0.076	2.328	0.020	-0.035	-2.193	0.028	25
ENSG00000164120	15-hydroxyprostaglandin dehydrogenase [Source:HGNC Symbol;Acc:HGNC:5154]	HPGD	protein_coding	-0.097	-2.205	0.027	0.162	3.025	0.002	30
ENSG00000178922	hydroxypyruvate isomerase (putative) [Source:HGNC Symbol;Acc:HGNC:26948]	HYI	protein_coding	-0.058	-2.057	0.040	0.057	3.382	0.001	28
ENSG00000244242	interferon induced transmembrane protein 10 [Source:HGNC Symbol;Acc:HGNC:40022]	IFITM10	protein_coding	0.140	2.381	0.017	-0.162	-2.105	0.035	28
ENSG00000128581	intraflagellar transport 22 [Source:HGNC Symbol;Acc:HGNC:21895]	IFT22	protein_coding	-0.039	-2.937	0.003	0.030	2.505	0.012	29
ENSG00000211970	immunoglobulin heavy variable 4-61 [Source:HGNC Symbol;Acc:HGNC:5655]	IGHV4-61	IG_V_gene	-0.148	-2.152	0.031	0.206	2.787	0.005	30
ENSG00000244116	immunoglobulin kappa variable 2-28 [Source:HGNC Symbol;Acc:HGNC:5783]	IGKV2-28	IG_V_gene	-0.228	-2.054	0.040	0.398	2.484	0.013	32
ENSG00000134470	interleukin 15 receptor subunit alpha [Source:HGNC Symbol;Acc:HGNC:5978]	IL15RA	protein_coding	0.072	2.361	0.018	-0.040	-2.321	0.020	25
ENSG0000008114	interphotoreceptor matrix	IMPG2	protein_coding	-0.093	-2.069	0.039	0.060	2.908	0.004	26

8	proteoglycan 2 [Source:HGNC Symbol;Acc:HGNC:18362]									
ENSG00000151689	inositol polyphosphate-1-phosphatase [Source:HGNC Symbol;Acc:HGNC:6071]	INPP1	protein_coding	-0.037	-2.037	0.042	0.029	2.260	0.024	29
ENSG00000169896	integrin subunit alpha M [Source:HGNC Symbol;Acc:HGNC:6149]	ITGAM	protein_coding	0.149	3.388	0.001	-0.057	-3.093	0.002	26
ENSG00000123700	potassium inwardly rectifying channel subfamily J member 2 [Source:HGNC Symbol;Acc:HGNC:6263]	KCNJ2	protein_coding	-0.142	-2.409	0.016	0.060	2.625	0.009	25
ENSG00000267365	KCNJ2 antisense RNA 1 [Source:HGNC Symbol;Acc:HGNC:43720]	KCNJ2-AS1	lncRNA	-0.117	-2.057	0.040	0.073	3.476	0.001	27
ENSG00000155666	lysine demethylase 8 [Source:HGNC Symbol;Acc:HGNC:25840]	KDM8	protein_coding	-0.085	-2.103	0.035	0.078	2.934	0.003	28
ENSG00000110427	KIAA1549 like [Source:HGNC Symbol;Acc:HGNC:24836]	KIAA1549L	protein_coding	0.272	3.271	0.001	-0.132	-2.755	0.006	26
ENSG00000165185	KIAA1958 [Source:HGNC Symbol;Acc:HGNC:23427]	KIAA1958	protein_coding	-0.090	-2.829	0.005	0.084	3.857	0.000	28
ENSG00000174010	kelch like family member 15 [Source:HGNC Symbol;Acc:HGNC:29347]	KLHL15	protein_coding	-0.099	-3.813	0.000	0.057	2.844	0.004	26
ENSG00000114796	kelch like family member 24 [Source:HGNC Symbol;Acc:HGNC:25947]	KLHL24	protein_coding	-0.037	-2.774	0.006	0.024	2.077	0.038	27
ENSG00000230658	KLHL7 divergent transcript [Source:HGNC Symbol;Acc:HGNC:43431]	KLHL7-DT	lncRNA	-0.279	-3.088	0.002	0.078	2.141	0.032	26
ENSG00000170484	keratin 74 [Source:HGNC Symbol;Acc:HGNC:28929]	KRT74	protein_coding	-0.150	-2.101	0.036	0.573	2.787	0.005	32
ENSG00000137944	kynurenine aminotransferase 3 [Source:HGNC Symbol;Acc:HGNC:33238]	KYAT3	protein_coding	-0.050	-2.300	0.021	0.052	2.433	0.015	30
ENSG00000188186	late endosomal/lysosomal adaptor, MAPK and MTOR activator 4 [Source:HGNC Symbol;Acc:HGNC:33772]	LAMTOR4	protein_coding	-0.022	-2.436	0.015	0.053	4.332	0.000	32
ENSG00000236090	lactate dehydrogenase A pseudogene 3 [Source:HGNC Symbol;Acc:HGNC:6538]	LDHAP3	processed_pseudogene	-0.175	-2.095	0.036	0.096	2.166	0.030	26
ENSG00000223935	LGALSL divergent transcript [Source:HGNC	LGALSL-DT	lncRNA	0.105	2.596	0.009	-0.409	-3.414	0.001	32

	Symbol;Acc:HGNC:53951]									
ENSG00000050405	LIM domain and actin binding 1 [Source:HGNC Symbol;Acc:HGNC:24636]	LIMA1	protein_coding	0.080	2.246	0.025	-0.016	-2.159	0.031	24
ENSG00000148943	lin-7 homolog C, crumbs cell polarity complex component [Source:HGNC Symbol;Acc:HGNC:17789]	LIN7C	protein_coding	-0.029	-2.115	0.034	0.026	2.026	0.043	28
ENSG00000223685	long intergenic non-protein coding RNA 571 [Source:HGNC Symbol;Acc:HGNC:43721]	LINC00571	lncRNA	-0.137	-2.796	0.005	0.107	3.486	0.000	28
ENSG00000223546	long intergenic non-protein coding RNA 630 [Source:HGNC Symbol;Acc:HGNC:44263]	LINC00630	lncRNA	0.248	5.960	0.000	-0.021	-2.093	0.036	24
ENSG00000281852	long intergenic non-protein coding RNA 891 [Source:HGNC Symbol;Acc:HGNC:48577]	LINC00891	lncRNA	-0.087	-2.084	0.037	0.030	1.999	0.046	25
ENSG00000235314	long intergenic non-protein coding RNA 957 [Source:HGNC Symbol;Acc:HGNC:22332]	LINC00957	lncRNA	-0.083	-2.562	0.010	0.021	2.039	0.041	24
ENSG00000261617	long intergenic non-protein coding RNA 2177 [Source:HGNC Symbol;Acc:HGNC:53039]	LINC02177	lncRNA	-0.194	-1.966	0.049	0.102	2.123	0.034	26
ENSG00000235160	long intergenic non-protein coding RNA 2248 [Source:HGNC Symbol;Acc:HGNC:53147]	LINC02248	lncRNA	-0.073	-2.045	0.041	0.027	2.296	0.022	27
ENSG00000251432	long intergenic non-protein coding RNA 2615 [Source:HGNC Symbol;Acc:HGNC:53402]	LINC02615	lncRNA	0.106	2.214	0.027	-0.134	-2.049	0.040	30
ENSG00000198121	lysophosphatidic acid receptor 1 [Source:HGNC Symbol;Acc:HGNC:3166]	LPAR1	protein_coding	-0.035	-2.648	0.008	0.059	5.651	0.000	32
ENSG00000148356	leucine rich repeat and sterile alpha motif containing 1 [Source:HGNC Symbol;Acc:HGNC:25135]	LRSAM1	protein_coding	0.036	2.159	0.031	-0.032	-2.162	0.031	29
ENSG00000180660	mab-21 like 1 [Source:HGNC Symbol;Acc:HGNC:6757]	MAB21L1	protein_coding	0.062	2.085	0.037	-0.221	-2.657	0.008	32
ENSG00000139625	mitogen-activated protein kinase kinase kinase 12 [Source:HGNC Symbol;Acc:HGNC:6851]	MAP3K12	protein_coding	0.055	2.577	0.010	-0.073	-3.675	0.000	29

ENSG00000156875	major facilitator superfamily domain containing 14A [Source:HGNC Symbol;Acc:HGNC:23363]	MFSD14A	protein_coding	-0.034	-2.177	0.029	0.022	2.253	0.024	28
ENSG00000154889	metallophosphoesterase 1 [Source:HGNC Symbol;Acc:HGNC:15988]	MPPE1	protein_coding	0.046	2.046	0.041	-0.025	-2.504	0.012	28
ENSG00000198727	mitochondrially encoded cytochrome b [Source:HGNC Symbol;Acc:HGNC:7427]	MT-CYB	protein_coding	0.056	2.485	0.013	-0.030	-2.170	0.030	26
ENSG00000132938	microtubule associated scaffold protein 2 [Source:HGNC Symbol;Acc:HGNC:20595]	MTUS2	protein_coding	0.034	2.135	0.033	-0.025	-1.961	0.050	29
ENSG00000205277	mucin 12, cell surface associated [Source:HGNC Symbol;Acc:HGNC:7510]	MUC12	protein_coding	-0.240	-2.255	0.024	0.388	3.648	0.000	30
ENSG00000013364	major vault protein [Source:HGNC Symbol;Acc:HGNC:7531]	MVP	protein_coding	0.042	2.057	0.040	-0.029	-1.962	0.050	27
ENSG00000151503	non-SMC condensin II complex subunit D3 [Source:HGNC Symbol;Acc:HGNC:28952]	NCAPD3	protein_coding	-0.080	-2.090	0.037	0.041	2.749	0.006	28
ENSG00000116701	neutrophil cytosolic factor 2 [Source:HGNC Symbol;Acc:HGNC:7661]	NCF2	protein_coding	-0.069	-2.871	0.004	0.109	2.438	0.015	31
ENSG00000145912	NHP2 ribonucleoprotein [Source:HGNC Symbol;Acc:HGNC:14377]	NHP2	protein_coding	-0.047	-3.112	0.002	0.030	2.838	0.005	28
ENSG00000048162	NOP16 nucleolar protein [Source:HGNC Symbol;Acc:HGNC:26934]	NOP16	protein_coding	0.061	3.355	0.001	-0.037	-2.866	0.004	29
ENSG00000185551	nuclear receptor subfamily 2 group F member 2 [Source:HGNC Symbol;Acc:HGNC:7976]	NR2F2	protein_coding	0.018	2.178	0.029	-0.038	-2.568	0.010	32
ENSG00000179915	neurexin 1 [Source:HGNC Symbol;Acc:HGNC:8008]	NRXN1	protein_coding	-0.075	-2.125	0.034	0.039	2.334	0.020	25
ENSG00000173598	nudix hydrolase 4 [Source:HGNC Symbol;Acc:HGNC:8051]	NUDT4	protein_coding	-0.057	-2.542	0.011	0.021	2.317	0.021	25
ENSG00000111331	2'-5'-oligoadenylate synthetase 3 [Source:HGNC Symbol;Acc:HGNC:8088]	OAS3	protein_coding	0.062	2.164	0.030	-0.074	-2.216	0.027	28
ENSG00000116774	olfactomedin like 3 [Source:HGNC	OLFML3	protein_coding	-0.040	-2.043	0.041	0.027	2.016	0.044	29

	Symbol;Acc:HGNC:24956]									
ENSG00000083093	partner and localizer of BRCA2 [Source:HGNC Symbol;Acc:HGNC:26144]	PALB2	protein_coding	-0.061	-2.462	0.014	0.023	2.090	0.037	26
ENSG00000100105	POZ/BTB and AT hook containing zinc finger 1 [Source:HGNC Symbol;Acc:HGNC:13071]	PATZ1	protein_coding	0.020	2.096	0.036	-0.039	-2.541	0.011	32
ENSG00000204304	PBX homeobox 2 [Source:HGNC Symbol;Acc:HGNC:8633]	PBX2	protein_coding	0.021	2.182	0.029	-0.020	-2.286	0.022	32
ENSG00000224729	PCOLCE antisense RNA 1 [Source:HGNC Symbol;Acc:HGNC:40430]	PCOLCE-AS1	lncRNA	0.135	2.145	0.032	-0.106	-2.189	0.029	29
ENSG00000115257	proprotein convertase subtilisin/kexin type 4 [Source:HGNC Symbol;Acc:HGNC:8746]	PCSK4	protein_coding	0.209	2.298	0.022	-0.081	-2.282	0.022	25
ENSG00000184588	phosphodiesterase 4B [Source:HGNC Symbol;Acc:HGNC:8781]	PDE4B	protein_coding	-0.040	-2.055	0.040	0.093	3.034	0.002	30
ENSG00000229828	PDE4DIP pseudogene 1 [Source:HGNC Symbol;Acc:HGNC:50867]	PDE4DIPP1	unprocessed_pseudogene	0.247	2.339	0.019	-0.305	-2.827	0.005	28
ENSG00000088356	p53 and DNA damage regulated 1 [Source:HGNC Symbol;Acc:HGNC:16119]	PDRG1	protein_coding	0.032	2.167	0.030	-0.082	-2.017	0.044	32
ENSG00000172367	PDZ domain containing 3 [Source:HGNC Symbol;Acc:HGNC:19891]	PDZD3	protein_coding	0.382	2.184	0.029	-0.198	-2.034	0.042	26
ENSG00000142655	peroxisomal biogenesis factor 14 [Source:HGNC Symbol;Acc:HGNC:8856]	PEX14	protein_coding	0.056	1.970	0.049	-0.022	-2.763	0.006	26
ENSG00000204220	prefoldin subunit 6 [Source:HGNC Symbol;Acc:HGNC:4926]	PFDN6	protein_coding	-0.074	-2.811	0.005	0.050	2.726	0.006	27
ENSG00000103066	phospholipase A2 group XV [Source:HGNC Symbol;Acc:HGNC:17163]	PLA2G15	protein_coding	0.033	2.294	0.022	-0.046	-2.650	0.008	27
ENSG00000189266	proline rich nuclear receptor coactivator 2 [Source:HGNC Symbol;Acc:HGNC:23158]	PNRC2	protein_coding	-0.020	-2.365	0.018	0.042	3.369	0.001	30
ENSG00000105568	protein phosphatase 2 scaffold subunit Aalpha [Source:HGNC Symbol;Acc:HGNC:9302]	PPP2R1A	protein_coding	-0.034	-2.310	0.021	0.018	2.034	0.042	26

ENSG00000138738	PR/SET domain 5 [Source:HGNC Symbol;Acc:HGNC:9349]	PRDM5	protein_coding	-0.084	-2.881	0.004	0.031	2.072	0.038	27
ENSG00000204540	psoriasis susceptibility 1 candidate 1 [Source:HGNC Symbol;Acc:HGNC:17202]	PSORS1C1	protein_coding	0.183	2.085	0.037	-0.075	-2.113	0.035	28
ENSG00000134222	proline and serine rich coiled-coil 1 [Source:HGNC Symbol;Acc:HGNC:24472]	PSRC1	protein_coding	0.069	2.553	0.011	-0.118	-4.530	0.000	31
ENSG00000185920	patched 1 [Source:HGNC Symbol;Acc:HGNC:9585]	PTCH1	protein_coding	0.058	2.134	0.033	-0.102	-2.557	0.011	31
ENSG00000237984	phosphatase and tensin homolog pseudogene 1 [Source:HGNC Symbol;Acc:HGNC:9589]	PTENP1	transcribed_processed_pseudogene	-0.106	-2.322	0.020	0.114	3.504	0.000	28
ENSG00000099246	RAB18, member RAS oncogene family [Source:HGNC Symbol;Acc:HGNC:14244]	RAB18	protein_coding	-0.034	-2.695	0.007	0.014	2.310	0.021	26
ENSG00000172007	RAB33B, member RAS oncogene family [Source:HGNC Symbol;Acc:HGNC:16075]	RAB33B	protein_coding	-0.055	-2.425	0.015	0.034	5.120	0.000	26
ENSG00000136144	RCC1 and BTB domain containing protein 1 [Source:HGNC Symbol;Acc:HGNC:18243]	RCBTB1	protein_coding	0.026	2.043	0.041	-0.024	-2.551	0.011	29
ENSG00000159788	regulator of G protein signaling 12 [Source:HGNC Symbol;Acc:HGNC:9994]	RGS12	protein_coding	0.086	3.538	0.000	-0.049	-2.477	0.013	28
ENSG00000091844	regulator of G protein signaling 17 [Source:HGNC Symbol;Acc:HGNC:14088]	RGS17	protein_coding	-0.122	-2.325	0.020	0.142	2.405	0.016	29
ENSG00000229927	RHEB pseudogene 1 [Source:HGNC Symbol;Acc:HGNC:10010]	RHEBP1	processed_pseudogene	0.244	2.712	0.007	-0.284	-3.427	0.001	28
ENSG00000140983	ras homolog family member T2 [Source:HGNC Symbol;Acc:HGNC:21169]	RHOT2	protein_coding	0.046	2.651	0.008	-0.071	-2.912	0.004	29
ENSG00000255794	rhabdomyosarcoma 2 associated transcript [Source:HGNC Symbol;Acc:HGNC:29893]	RMST	lncRNA	-0.107	-2.226	0.026	0.055	2.594	0.009	28
ENSG00000265727	RNA, 7SL, cytoplasmic 648, pseudogene [Source:HGNC Symbol;Acc:HGNC:46664]	RN7SL648P	misc_RNA	0.123	1.979	0.048	-0.082	-2.357	0.018	26
ENSG0000013387	ring finger protein 122	RNF122	protein_coding	-0.038	-1.973	0.049	0.067	1.990	0.047	28

4	[Source:HGNC Symbol;Acc:HGNC:21147]									
ENSG00000154133	roundabout guidance receptor 4 [Source:HGNC Symbol;Acc:HGNC:17985]	ROBO4	protein_coding	0.061	2.200	0.028	-0.061	-2.235	0.025	28
ENSG00000100316	ribosomal protein L3 [Source:HGNC Symbol;Acc:HGNC:10332]	RPL3	protein_coding	-0.025	-2.644	0.008	0.025	2.764	0.006	29
ENSG00000174444	ribosomal protein L4 [Source:HGNC Symbol;Acc:HGNC:10353]	RPL4	protein_coding	-0.028	-2.526	0.012	0.019	2.127	0.033	29
ENSG00000089009	ribosomal protein L6 [Source:HGNC Symbol;Acc:HGNC:10362]	RPL6	protein_coding	-0.016	-2.321	0.020	0.016	2.021	0.043	29
ENSG00000160208	ribosomal RNA processing 1B [Source:HGNC Symbol;Acc:HGNC:23818]	RRP1B	protein_coding	-0.048	-2.913	0.004	0.017	2.667	0.008	26
ENSG00000134321	radical S-adenosyl methionine domain containing 2 [Source:HGNC Symbol;Acc:HGNC:30908]	RSAD2	protein_coding	0.103	2.971	0.003	-0.079	-2.171	0.030	28
ENSG00000167524	ribosomal protein S6 kinase related [Source:HGNC Symbol;Acc:HGNC:26314]	RSKR	protein_coding	0.085	2.049	0.041	-0.052	-2.328	0.020	29
ENSG00000182552	RWD domain containing 4 [Source:HGNC Symbol;Acc:HGNC:23750]	RWDD4	protein_coding	0.035	2.823	0.005	-0.024	-2.384	0.017	28
ENSG00000163785	receptor like tyrosine kinase [Source:HGNC Symbol;Acc:HGNC:10481]	RYK	protein_coding	-0.072	-2.830	0.005	0.018	2.079	0.038	25
ENSG00000246273	SBF2 antisense RNA 1 [Source:HGNC Symbol;Acc:HGNC:27438]	SBF2-AS1	lncRNA	-0.096	-2.650	0.008	0.111	2.543	0.011	28
ENSG00000166562	SEC11 homolog C, signal peptidase complex subunit [Source:HGNC Symbol;Acc:HGNC:23400]	SEC11C	protein_coding	-0.054	-3.153	0.002	0.025	2.145	0.032	27
ENSG00000162430	selenoprotein N [Source:HGNC Symbol;Acc:HGNC:15999]	SELENON	protein_coding	0.047	2.210	0.027	-0.020	-2.512	0.012	25
ENSG00000197417	sedoheptulokinase [Source:HGNC Symbol;Acc:HGNC:1492]	SHPK	protein_coding	0.084	2.976	0.003	-0.030	-2.312	0.021	25
ENSG00000204351	Ski2 like RNA helicase [Source:HGNC Symbol;Acc:HGNC:10898]	SKIV2L	protein_coding	0.042	2.619	0.009	-0.061	-2.458	0.014	30

ENSG00000117090	signaling lymphocytic activation molecule family member 1 [Source:HGNC Symbol;Acc:HGNC:10903]	SLAMF1	protein_coding	0.346	3.717	0.000	-0.135	-2.122	0.034	26
ENSG00000151012	solute carrier family 7 member 11 [Source:HGNC Symbol;Acc:HGNC:11059]	SLC7A11	protein_coding	-0.139	-2.700	0.007	0.109	2.683	0.007	28
ENSG00000137571	solute carrier organic anion transporter family member 5A1 [Source:HGNC Symbol;Acc:HGNC:19046]	SLCO5A1	protein_coding	0.040	2.048	0.041	-0.053	-2.367	0.018	29
ENSG00000213599	SLX1A-SULT1A3 readthrough (NMD candidate) [Source:HGNC Symbol;Acc:HGNC:44437]	SLX1A-SULT1A3	lncRNA	0.248	2.067	0.039	-0.210	-1.960	0.050	26
ENSG00000170365	SMAD family member 1 [Source:HGNC Symbol;Acc:HGNC:6767]	SMAD1	protein_coding	0.088	2.263	0.024	-0.071	-2.621	0.009	28
ENSG00000198952	SMG5 nonsense mediated mRNA decay factor [Source:HGNC Symbol;Acc:HGNC:24644]	SMG5	protein_coding	0.021	2.334	0.020	-0.044	-4.206	0.000	32
ENSG00000172594	sphingomyelin phosphodiesterase acid like 3A [Source:HGNC Symbol;Acc:HGNC:17389]	SMPDL3A	protein_coding	-0.034	-2.577	0.010	0.048	2.574	0.010	32
ENSG00000102172	spermine synthase [Source:HGNC Symbol;Acc:HGNC:11123]	SMS	protein_coding	-0.064	-3.330	0.001	0.019	2.091	0.037	26
ENSG00000100028	small nuclear ribonucleoprotein D3 polypeptide [Source:HGNC Symbol;Acc:HGNC:11160]	SNRPD3	protein_coding	-0.043	-2.314	0.021	0.030	2.169	0.030	27
ENSG00000110025	sorting nexin 15 [Source:HGNC Symbol;Acc:HGNC:14978]	SNX15	protein_coding	-0.045	-2.048	0.041	0.056	2.514	0.012	29
ENSG00000109610	superoxide dismutase 3 [Source:HGNC Symbol;Acc:HGNC:11181]	SOD3	protein_coding	0.045	2.078	0.038	-0.042	-2.947	0.003	28
ENSG00000067066	SP100 nuclear antigen [Source:HGNC Symbol;Acc:HGNC:11206]	SP100	protein_coding	0.031	2.612	0.009	-0.016	-2.406	0.016	28
ENSG00000122432	spermatogenesis associated 1 [Source:HGNC Symbol;Acc:HGNC:14682]	SPATA1	protein_coding	-0.177	-3.427	0.001	0.132	2.790	0.005	28
ENSG0000018941	spermatogenesis associated 41	SPATA41	lncRNA	0.130	2.321	0.020	-0.216	-2.248	0.025	28

9	[Source:HGNC Symbol;Acc:HGNC:48613]									
ENSG00000116096	sepiapterin reductase [Source:HGNC Symbol;Acc:HGNC:11257]	SPR	protein_coding	0.033	1.979	0.048	-0.030	-2.199	0.028	28
ENSG00000277363	SRC kinase signaling inhibitor 1 [Source:HGNC Symbol;Acc:HGNC:29506]	SRCIN1	protein_coding	-0.115	-2.177	0.029	0.123	3.402	0.001	28
ENSG00000008513	ST3 beta-galactoside alpha-2,3-sialyltransferase 1 [Source:HGNC Symbol;Acc:HGNC:10862]	ST3GAL1	protein_coding	0.018	2.240	0.025	-0.068	-5.242	0.000	32
ENSG00000144057	ST6 beta-galactoside alpha-2,6-sialyltransferase 2 [Source:HGNC Symbol;Acc:HGNC:10861]	ST6GAL2	protein_coding	0.183	2.772	0.006	-0.213	-2.549	0.011	29
ENSG00000160408	ST6 N-acetylgalactosaminide alpha-2,6-sialyltransferase 6 [Source:HGNC Symbol;Acc:HGNC:23364]	ST6GALNAC6	protein_coding	-0.046	-3.403	0.001	0.020	2.314	0.021	28
ENSG00000213533	STIM activating enhancer [Source:HGNC Symbol;Acc:HGNC:30526]	STIMATE	protein_coding	0.156	3.778	0.000	-0.048	-2.353	0.019	26
ENSG00000162520	syncoilin, intermediate filament protein [Source:HGNC Symbol;Acc:HGNC:28897]	SYNC	protein_coding	0.058	2.365	0.018	-0.039	-2.381	0.017	29
ENSG00000177156	transaldolase 1 [Source:HGNC Symbol;Acc:HGNC:11559]	TALDO1	protein_coding	-0.023	-2.168	0.030	0.042	3.088	0.002	29
ENSG00000138336	tet methylcytosine dioxygenase 1 [Source:HGNC Symbol;Acc:HGNC:29484]	TET1	protein_coding	-0.031	-2.086	0.037	0.025	2.089	0.037	28
ENSG00000174796	THAP domain containing 6 [Source:HGNC Symbol;Acc:HGNC:23189]	THAP6	protein_coding	0.043	2.154	0.031	-0.021	-1.993	0.046	28
ENSG00000144229	thrombospondin type 1 domain containing 7B [Source:HGNC Symbol;Acc:HGNC:29348]	THSD7B	protein_coding	0.099	2.389	0.017	-0.142	-2.700	0.007	32
ENSG00000066654	THUMP domain containing 1 [Source:HGNC Symbol;Acc:HGNC:23807]	THUMPD1	protein_coding	-0.044	-2.314	0.021	0.032	2.156	0.031	28
ENSG00000163659	TCDD inducible poly(ADP-ribose) polymerase [Source:HGNC Symbol;Acc:HGNC:23696]	TIPARP	protein_coding	-0.027	-2.789	0.005	0.097	2.822	0.005	32
ENSG0000022882	tousled like kinase 2	TLK2P2	processed_pseudogene	0.108	2.520	0.012	-0.209	-3.340	0.001	32

8	pseudogene 2 [Source:HGNC Symbol;Acc:HGNC:22227]									
ENSG00000164124	transmembrane protein 144 [Source:HGNC Symbol;Acc:HGNC:25633]	TMEM144	protein_coding	-0.042	-2.752	0.006	0.032	2.808	0.005	27
ENSG00000163444	transmembrane protein 183A [Source:HGNC Symbol;Acc:HGNC:20173]	TMEM183A	protein_coding	0.066	2.038	0.042	-0.020	-2.656	0.008	26
ENSG00000187049	transmembrane protein 216 [Source:HGNC Symbol;Acc:HGNC:25018]	TMEM216	protein_coding	-0.063	-2.281	0.023	0.063	2.364	0.018	31
ENSG00000205084	transmembrane protein 231 [Source:HGNC Symbol;Acc:HGNC:37234]	TMEM231	protein_coding	-0.074	-2.893	0.004	0.062	3.333	0.001	29
ENSG00000134490	transmembrane protein 241 [Source:HGNC Symbol;Acc:HGNC:31723]	TMEM241	protein_coding	0.110	2.376	0.018	-0.095	-2.965	0.003	28
ENSG00000182087	transmembrane protein 259 [Source:HGNC Symbol;Acc:HGNC:17039]	TMEM259	protein_coding	0.034	3.097	0.002	-0.012	-2.021	0.043	25
ENSG00000121858	TNF superfamily member 10 [Source:HGNC Symbol;Acc:HGNC:11925]	TNFSF10	protein_coding	0.047	2.603	0.009	-0.038	-2.377	0.017	28
ENSG00000118194	troponin T2, cardiac type [Source:HGNC Symbol;Acc:HGNC:11949]	TNNT2	protein_coding	-0.047	-2.064	0.039	0.027	2.082	0.037	25
ENSG00000111077	tensin 2 [Source:HGNC Symbol;Acc:HGNC:19737]	TNS2	protein_coding	0.069	2.118	0.034	-0.027	-1.978	0.048	26
ENSG00000177302	DNA topoisomerase III alpha [Source:HGNC Symbol;Acc:HGNC:11992]	TOP3A	protein_coding	-0.073	-2.714	0.007	0.072	3.268	0.001	28
ENSG00000166166	tRNA methyltransferase 61A [Source:HGNC Symbol;Acc:HGNC:23790]	TRMT61A	protein_coding	-0.047	-2.395	0.017	0.039	2.291	0.022	28
ENSG00000179981	teashirt zinc finger homeobox 1 [Source:HGNC Symbol;Acc:HGNC:10669]	TSHZ1	protein_coding	0.029	2.041	0.041	-0.027	-1.985	0.047	29
ENSG00000160803	ubiquilin 4 [Source:HGNC Symbol;Acc:HGNC:1237]	UBQLN4	protein_coding	0.023	2.140	0.032	-0.053	-3.550	0.000	32
ENSG00000175970	unc-119 lipid binding chaperone B [Source:HGNC Symbol;Acc:HGNC:16488]	UNC119B	protein_coding	0.046	2.336	0.019	-0.021	-1.986	0.047	26
ENSG00000132478	unk zinc finger [Source:HGNC Symbol;Acc:HGNC:29369]	UNK	protein_coding	0.048	2.024	0.043	-0.027	-2.183	0.029	27
ENSG0000016906	UPF3A regulator of nonsense	UPF3A	protein_coding	0.029	1.983	0.047	-0.040	-1.987	0.047	32

2	mediated mRNA decay [Source:HGNC Symbol;Acc:HGNC:20332]									
ENSG00000136878	ubiquitin specific peptidase 20 [Source:HGNC Symbol;Acc:HGNC:12619]	USP20	protein_coding	0.060	2.276	0.023	-0.027	-2.252	0.024	26
ENSG00000234769	WASP family homolog 4, pseudogene [Source:HGNC Symbol;Acc:HGNC:14126]	WASH4P	unprocessed_pseudogene	-0.326	-3.616	0.000	0.163	2.890	0.004	27
ENSG00000164961	WASH complex subunit 5 [Source:HGNC Symbol;Acc:HGNC:28984]	WASHC5	protein_coding	0.071	2.519	0.012	-0.042	-2.585	0.010	28
ENSG00000248334	WAS protein homolog associated with actin, golgi membranes and microtubules pseudogene 2 [Source:HGNC Symbol;Acc:HGNC:32360]	WHAMMP2	transcribed_unprocessed_pseudogene	0.074	2.566	0.010	-0.062	-2.701	0.007	28
ENSG00000152422	X-ray repair cross complementing 4 [Source:HGNC Symbol;Acc:HGNC:12831]	XRCC4	protein_coding	0.051	2.884	0.004	-0.037	-2.044	0.041	29
ENSG00000239407	novel transcript	Z68871.1	lncRNA	-0.132	-3.035	0.002	0.034	1.971	0.049	25
ENSG00000166707	zinc finger CCHC-type containing 18 [Source:HGNC Symbol;Acc:HGNC:32459]	ZCCHC18	protein_coding	-0.166	-1.998	0.046	0.153	2.229	0.026	29
ENSG00000160445	zyg-11 related cell cycle regulator [Source:HGNC Symbol;Acc:HGNC:30960]	ZER1	protein_coding	0.037	2.250	0.024	-0.018	-2.405	0.016	26
ENSG00000066827	zinc finger and AT-hook domain containing [Source:HGNC Symbol;Acc:HGNC:19899]	ZFAT	protein_coding	0.057	2.029	0.042	-0.033	-2.076	0.038	27
ENSG00000275111	zinc finger protein 2 [Source:HGNC Symbol;Acc:HGNC:12991]	ZNF2	protein_coding	-0.075	-2.204	0.028	0.046	2.863	0.004	27
ENSG00000166261	zinc finger protein 202 [Source:HGNC Symbol;Acc:HGNC:12994]	ZNF202	protein_coding	-0.058	-1.971	0.049	0.090	2.635	0.008	30
ENSG00000198026	zinc finger protein 335 [Source:HGNC Symbol;Acc:HGNC:15807]	ZNF335	protein_coding	0.030	2.648	0.008	-0.049	-2.581	0.010	32
ENSG00000198597	zinc finger protein 536 [Source:HGNC Symbol;Acc:HGNC:29025]	ZNF536	protein_coding	0.060	2.325	0.020	-0.094	-2.165	0.030	30
ENSG0000017822	zinc finger protein 543	ZNF543	protein_coding	-0.029	-2.066	0.039	0.026	2.346	0.019	28

9	[Source:HGNC Symbol;Acc:HGNC:25281]									
ENSG00000230844	ZNF674 antisense RNA 1 (head to head) [Source:HGNC Symbol;Acc:HGNC:44266]	ZNF674-AS1	lncRNA	-0.095	-2.285	0.022	0.044	2.225	0.026	26
Metabolites		a-ketoglutarate	metabolite	-0.049	-0.787	0.431	0.045	1.408	0.159	29
		Carnitine C12	metabolite	0.018	0.131	0.896	0.077	2.195	0.028	25
		Carnitine C14:1	metabolite	0.020	0.233	0.816	0.089	2.791	0.005	25
		Carnitine C16	metabolite	0.041	0.933	0.351	0.177	4.782	0.000	32
		Carnitine C16-OH	metabolite	0.042	1.102	0.270	0.169	1.693	0.090	32
		Carnitine C18	metabolite	0.076	0.747	0.455	0.067	2.672	0.008	25
		Carnitine C18-OH	metabolite	-0.055	-0.575	0.565	0.086	2.467	0.014	24
		Carnitine C18:1	metabolite	0.043	0.451	0.652	0.053	1.591	0.112	26
		Carnitine C18:1-OH	metabolite	-0.008	-0.080	0.936	0.094	3.605	0.000	24
		Carnitine C18:2	metabolite	0.059	0.625	0.532	0.040	1.042	0.297	26
		Carnitine C18:2-OH	metabolite	0.047	1.104	0.270	0.224	3.257	0.001	32
		Glutarylcarnitine (C5DC)	metabolite	-0.024	-0.163	0.871	0.062	1.484	0.138	25
		Carnitine C8:1	metabolite	0.063	1.095	0.273	0.027	0.359	0.719	30
		Ribose-5-phosphate	metabolite	0.124	2.138	0.033	0.218	2.416	0.016	32

Table S3 - Pre- and Post-operative characteristics in samples with transcriptomics and metabolomics analyses. (*) - Tests among BMI groups were conducted by exact test for categorical variables, and ANOVA or non-parametric Kruskal-Wallis test for continuous variables. Data are presented as n (%) for categorical variables and mean (SD) or median (IQR) for continuous variables. Abbreviations: ACE – Angiotensin Converting Enzyme; AKI – Acute Kidney Injury; BMI – Body Mass Index; CABG – Coronary artery Bypass Grafting; CCS – Canadian Cardiovascular Society; Hct – Haematocrit; FiO2 – Fraction of Inspired Oxygen; KDIGO - The Kidney Disease Improving Global Outcomes; MABP – Mean Arterial Blood Pressure; MODS – Multiorgan Dysfunction Syndrome; NYHA – New York Heart Association; PO2 – Partial Pressure of Oxygen; RBC – Red Blood Cells; VD – Vessel Disease.

Subset (n=60)	BMI<25 (n=16)	25≤BMI≤32 (n=33)	BMI>32 (n=11)	p-value	Missing data (n)
Age (years) - Median (1st - 3rd quartile)	67 (62 - 75)	67 (61.8 - 74)	60 (54 - 67.5)	0.415	0
Sex (male) - n (%)	14 (88%)	28 (85%)	10 (91%)	1.000	0
Ethnic (White) - n (%)	14 (88%)	28 (85%)	10 (91%)	0.336	0
BMI	67 (62 - 75)	67 (61.8 - 74)	60 (54 - 67.5)	<0.001	0
Smoking History					
Never smoker - n (%)	6 (38%)	14 (42%)	4 (36%)	0.944	0
Ex-smoker - n (%)	8 (50%)	16 (48%)	5 (45%)		
Current smoker - n (%)	2 (12%)	3 (9%)	2 (18%)		
**Medical history**					
Diabetes - n (%)	2 (12%)	9 (27%)	4 (36%)	0.342	0
Permanent Pacemaker - n (%)	1 (6%)	1 (3%)	0 (0%)	1	0
Stroke/Transient Ischaemic Attack - n (%)	2 (12%)	3 (9%)	1 (9%)	1	0
Chronic pulmonary disease - n (%)	3 (19%)	3 (9%)	2 (18%)	0.516	0
Neurological disease - n (%)	0 (0%)	0 (0%)	0 (0%)	--	0
Renal disease - n (%)	0 (0%)	1 (3%)	2 (18%)	0.139	0
Myocardial infarction - n (%)	5 (31%)	8 (24%)	1 (9%)	0.464	0
Extracardiac arteriopathy - n (%)	2 (12%)	4 (12%)	1 (9%)	1	0
Liver disease - n (%)	0 (0%)	0 (0%)	0 (0%)	--	0
Pulmonary hypertension - n (%)	0 (0%)	1 (3%)	0 (0%)	1	0
**Pre-operative Medication Histroy**					
Statin - n (%)	12 (75%)	23 (70%)	10 (91%)	0.476	0
Anti-platelet agents - n (%)	11 (69%)	28 (85%)	10 (91%)	0.354	0
ACE inhibitors - n (%)	9 (56%)	14 (42%)	5 (45%)	0.691	0
**Clinical characteristics**					
Surgery type					
CABG only - n (%)	13 (81%)	29 (88%)	10 (91%)	0.772	0
CABG & Valve - n (%)	3 (19%)	4 (12%)	1 (9%)		
NYHA					
Class I - n (%)	4 (25%)	10 (30%)	4 (36%)	0.773	0
Class II - n (%)	11 (69%)	20 (61%)	5 (45%)		
Class III, IV - n (%)	1 (6%)	3 (9%)	2 (18%)		
CCS					
Asymptomatic - n (%)	5 (31%)	2 (6%)	2 (18%)	0.341	0

Class I - n (%)	6 (38%)	11 (33%)	4 (36%)		
Class II - n (%)	4 (25%)	16 (48%)	4 (36%)		
Class III, IV - n (%)	1 (6%)	4 (12%)	1 (9%)		
Left Ventricular Ejection Fraction					
Good (>49%) - n (%)	13 (81%)	27 (82%)	8 (73%)	0.827	0
Fair (30-49%) - n (%)	3 (19%)	6 (18%)	3 (27%)		
Left main stem disease - n (%)	2 (12%)	9 (27%)	2 (18%)	0.535	0
Extent of coronary disease					
Normal/ 1VD - n (%)	1 (6%)	1 (3%)	3 (27%)	0.04	0
2VD - n (%)	7 (44%)	6 (18%)	2 (18%)	0.04	0
3VD - n (%)	8 (50%)	26 (79%)	6 (55%)	0.04	0
Pre-operative PaO2/FiO2 ratio - Median (1st - 3rd quartile)	533 (445.2 - 690.5)	495 (409.5 - 533.3)	457 (409.5 - 457.1)	0.280	8
Pre-operative Platelets count (x109/L) - Mean (STD)	222.2 (55.7)	232.6 (64.9)	231.5 (63.3)	0.858	1
Pre-operative Serum Creatinine (umol/L) - Median (1st - 3rd quartile)	78 (70.8 - 102.5)	77 (68 - 87)	84 (77 - 92)	0.491	0
Pre-operative Bilirubin (umol/L) - Median (1st - 3rd quartile)	10 (7.5 - 12.5)	11 (8.5 - 12.5)	8 (8 - 12)	0.801	5
**Postoperative**					
Hct (%) - Mean (STD)	31.9 (3.9)	34.6 (3.9)	35 (4.9)	0.076	0
MABP (mm Hg) - Median (1st - 3rd quartile)	76 (68.8 - 85)	73 (64 - 76)	65 (61.5 - 72)	0.672	0
Lactate (mmol/L) - Median (1st - 3rd quartile)	2 (1.1 - 2)	2 (1.4 - 2.2)	2 (1.3 - 2)	0.970	1
Inotropic score at 24h - Median (1st - 3rd quartile)	0 (0 - 2)	1 (0 - 3)	0 (0 - 3)	0.198	7
Vasoactive score at 24h - Median (1st - 3rd quartile)	5 (2.5 - 9)	5 (2.5 - 7)	4 (2 - 7.5)	0.198	4
MODS ICU - Median (1st - 3rd quartile)	1 (1 - 3)	2 (2 - 3)	3 (2 - 3)	**0.044**	2
Worst postoperative MODS score - Median (1st - 3rd quartile)	2 (1 - 5)	3 (2 - 4)	3 (3 - 4)	**0.023**	1
PaO2/FiO2 ratio at 48h - Median (1st - 3rd quartile)	410 (342.1 - 572.6)	395 (307.1 - 409.5)	410 (342.3 - 457.1)	0.127	1
Serum creatinine 48h (umol/L) - Median (1st - 3rd quartile)	73 (67.5 - 90)	71 (60 - 82)	74 (72 - 76)	0.383	2
RBC transfused postoperative - n (%)	8 (50%)	12 (36%)	2 (18%)	0.274	0
nonRBC transfusion at more than 48h - n (%)	2 (12%)	4 (12%)	1 (9%)	1	0
nonRBC transfusion within 48h - n (%)	6 (38%)	6 (18%)	0 (0%)	**0.046**	1
PaO2/FiO2 ratio at 48hr <=300 - n (%)	3 (19%)	4 (12%)	2 (18%)	0.7	1
AKI according to kdigo criteria - n (%)	1 (6%)	1 (3%)	0 (0%)	1	1

Table S4 – Details of the pathway analysis. Number of genes indicates number of transcripts in the analyzed dataset. Direction indicates gene set's up or downregulation.

Pathway	25≤BMI≤32 vs BMI<25				BMI>32 vs BMI<25				BMI>32 vs 25≤BMI≤32			
	Number of Genes	Direction	p-value	Adjusted p-value	Number of Genes	Direction	p-value	Adjusted p-value	Number of Genes	Direction	p-value	Adjusted p-value
Metabolism of amino acids and derivatives	327	Down	0.001	0.034	NA	NA	NA	NA	NA	NA	NA	NA
Translation	292	Down	0.000	0.004	NA	NA	NA	NA	NA	NA	NA	NA
Signaling by ROBO receptors	207	Down	0.000	0.004	207	Down	5.8E-04	0.034	NA	NA	NA	NA
rRNA processing	203	Down	0.000	0.000	203	Down	7.2E-05	0.006	NA	NA	NA	NA
rRNA processing in the nucleus and cytosol	191	Down	0.000	0.000	191	Down	3.1E-05	0.003	NA	NA	NA	NA
Major pathway of rRNA processing in the nucleolus and cytosol	181	Down	0.000	0.000	181	Down	1.5E-05	0.002	NA	NA	NA	NA
Regulation of expression of SLITs and ROBOs	163	Down	0.000	0.000	163	Down	7.7E-05	0.006	NA	NA	NA	NA
Influenza Infection	154	Down	0.000	0.000	154	Down	2.4E-06	0.000	NA	NA	NA	NA
Influenza Viral RNA Transcription and Replication	135	Down	0.000	0.000	135	Down	1.5E-07	0.000	NA	NA	NA	NA
Cap-dependent Translation Initiation	118	Down	0.000	0.000	118	Down	9.4E-10	0.000	NA	NA	NA	NA
Eukaryotic Translation Initiation	118	Down	0.000	0.000	118	Down	9.4E-10	0.000	NA	NA	NA	NA
Nonsense Mediated Decay (NMD) enhanced by the Exon Junction Complex (EJC)	114	Down	0.000	0.000	114	Down	1.1E-09	0.000	NA	NA	NA	NA
Nonsense-Mediated Decay (NMD)	114	Down	0.000	0.000	114	Down	1.1E-09	0.000	NA	NA	NA	NA
Selenoamino acid metabolism	114	Down	0.000	0.000	114	Down	1.2E-11	0.000	NA	NA	NA	NA
GTP hydrolysis and joining of the 60S ribosomal subunit	111	Down	0.000	0.000	111	Down	6.2E-11	0.000	NA	NA	NA	NA
SRP-dependent cotranslational protein targeting to membrane	111	Down	0.000	0.000	111	Down	2.2E-12	0.000	NA	NA	NA	NA
L13a-mediated translational silencing of Ceruloplasmin expression	110	Down	0.000	0.000	110	Down	1.1E-11	0.000	NA	NA	NA	NA
Formation of a pool of free 40S subunits	100	Down	0.000	0.000	100	Down	2.8E-13	0.000	NA	NA	NA	NA
Response of EIF2AK4 (GCN2) to amino acid deficiency	100	Down	0.000	0.000	100	Down	4.2E-13	0.000	NA	NA	NA	NA

Nonsense Mediated Decay (NMD) independent of the Exon Junction Complex (EJC)	94	Down	0.000	0.000	94	Down	3.1E-14	0.000	NA	NA	NA	NA
Eukaryotic Translation Elongation	92	Down	0.000	0.000	92	Down	1.1E-14	0.000	NA	NA	NA	NA
Eukaryotic Translation Termination	92	Down	0.000	0.000	92	Down	2.3E-13	0.000	NA	NA	NA	NA
Selenocysteine synthesis	92	Down	0.000	0.000	92	Down	3.1E-14	0.000	NA	NA	NA	NA
Peptide chain elongation	88	Down	0.000	0.000	88	Down	6.9E-15	0.000	NA	NA	NA	NA
Viral mRNA Translation	88	Down	0.000	0.000	88	Down	5.7E-15	0.000	NA	NA	NA	NA
Activation of the mRNA upon binding of the cap-binding complex and eIFs, and subsequent binding to 43S	59	Down	0.000	0.000	59	Down	1.3E-05	0.001	NA	NA	NA	NA
Ribosomal scanning and start codon recognition	58	Down	0.000	0.000	58	Down	5.3E-05	0.005	NA	NA	NA	NA
Translation initiation complex formation	58	Down	0.000	0.000	58	Down	1.1E-05	0.001	NA	NA	NA	NA
Interferon alpha/beta signaling	56	Up	0.000	0.008	NA	NA	NA	NA	NA	NA	NA	NA
Formation of the ternary complex, and subsequently, the 43S complex	51	Down	0.000	0.000	51	Down	1.3E-06	0.000	NA	NA	NA	NA
Iron uptake and transport	50	Down	0.000	0.017	NA	NA	NA	NA	NA	NA	NA	NA
Striated Muscle Contraction	29	Up	0.000	0.004	29	Up	1.5E-04	0.012	NA	NA	NA	NA
Synthesis, secretion, and deacylation of Ghrelin	12	Down	0.000	0.011	NA	NA	NA	NA	NA	NA	NA	NA
mitochondrial fatty acid beta-oxidation of saturated fatty acids	10	Up	0.000	0.026	NA	NA	NA	NA	NA	NA	NA	NA
Defective F8 binding to von Willebrand factor	2	Up	0.000	0.017	NA	NA	NA	NA	NA	NA	NA	NA
Defective F8 cleavage by thrombin	2	Up	0.000	0.017	NA	NA	NA	NA	NA	NA	NA	NA
HSF1-dependent transactivation	NA	NA	NA	NA	35	Up	2.2E-04	0.014	NA	NA	NA	NA
Keratan sulfate degradation	NA	NA	NA	NA	12	Down	3.3E-04	0.021	NA	NA	NA	NA
Erythrocytes take up oxygen and release carbon dioxide	NA	NA	NA	NA	8	Up	6.8E-05	0.006	NA	NA	NA	NA
TNFR1-mediated ceramide production	NA	NA	NA	NA	6	Down	8.2E-04	0.046	NA	NA	NA	NA
Alternative complement activation	NA	NA	NA	NA	4	Down	4.6E-04	0.028	NA	NA	NA	NA
Ribonuclease P activity (lncRNA, Gene Ontology)	NA	NA	NA	NA	2	Down	2.1E-04	0.014	NA	NA	NA	NA
tRNA processing (lncRNA, Gene Ontology)	NA	NA	NA	NA	2	Down	2.1E-04	0.014	NA	NA	NA	NA
ribonuclease P complex (lncRNA, Gene Ontology)	NA	NA	NA	NA	2	Down	2.1E-	0.014	NA	NA	NA	NA

							04					
RNA phosphodiester bond hydrolysis, endonucleolytic (lncRNA, Gene Ontology)	NA	NA	NA	NA	2	Down	2.1E-04	0.014	NA	NA	NA	NA
Defective ACTH causes Obesity and Pro-opiomelanocortinin deficiency (POMCD)	NA	NA	NA	NA	1	Up	7.3E-04	0.042	NA	NA	NA	NA
Binding and Uptake of Ligands by Scavenger Receptors	NA	NA	NA	NA	NA	NA	NA	NA	83	Up	2.3E-05	5.6E-03
Nucleolus (lncRNA, Gene Ontology)	NA	NA	NA	NA	83	Down	1.9E-09	0.000	83	Down	4.0E-06	1.6E-03
FCGR3A-mediated IL10 synthesis	NA	NA	NA	NA	NA	NA	NA	NA	82	Up	8.8E-05	1.5E-02
RNA processing (lncRNA, Gene Ontology)	NA	NA	NA	NA	81	Down	1.1E-09	0.000	81	Down	2.8E-06	1.4E-03
Antigen activates B Cell Receptor (BCR) leading to generation of second messengers	NA	NA	NA	NA	NA	NA	NA	NA	74	Up	9.8E-06	3.1E-03
FCERI mediated Ca+2 mobilization	NA	NA	NA	NA	NA	NA	NA	NA	74	Up	1.6E-04	2.4E-02
Role of phospholipids in phagocytosis	NA	NA	NA	NA	NA	NA	NA	NA	70	Up	1.3E-04	2.0E-02
Role of LAT2/NTAL/LAB on calcium mobilization	NA	NA	NA	NA	NA	NA	NA	NA	59	Up	2.6E-05	5.8E-03
FCGR activation	NA	NA	NA	NA	58	Up	4.8E-04	0.029	58	Up	3.6E-07	2.4E-04
Creation of C4 and C2 activators	NA	NA	NA	NA	NA	NA	NA	NA	56	Up	1.0E-05	3.1E-03
Scavenging of heme from plasma	NA	NA	NA	NA	55	Up	2.0E-04	0.014	55	Up	3.9E-07	2.4E-04
Classical antibody-mediated complement activation	NA	NA	NA	NA	NA	NA	NA	NA	51	Up	1.4E-05	3.8E-03
CD22 mediated BCR regulation	NA	NA	NA	NA	50	Up	9.8E-05	0.008	50	Up	8.4E-08	1.7E-04
Cholesterol biosynthesis	NA	NA	NA	NA	NA	NA	NA	NA	24	Up	1.4E-07	1.7E-04
Acetylcholine regulates insulin secretion	NA	NA	NA	NA	NA	NA	NA	NA	10	Down	2.8E-04	4.0E-02
Metallothioneins bind metals	NA	NA	NA	NA	NA	NA	NA	NA	6	Up	5.7E-05	1.1E-02
Peptide hormone biosynthesis	NA	NA	NA	NA	NA	NA	NA	NA	5	Up	2.9E-05	5.9E-03

Table S5 – Details of genes that were highly variable between the BMI groups (p-value < 0.05) with weighted gene correlation networks membership.

Ensembl ID	Gene Symbol	Description	Biotype	25≤BMI≤32 vs BMI<25	BMI>32 vs BMI<25	BMI>32 vs25≤BMI≤ 32	AveExpr	F	P.Value	Network
ENSG00000008516	MMP25	matrix metallopeptidase 25 [Source:HGNC Symbol;Acc:HGNC:14246]	protein_coding	0.660	1.483	0.823	-0.840	3.588	0.034	black
ENSG00000044524	EPHA3	EPH receptor A3 [Source:HGNC Symbol;Acc:HGNC:3387]	protein_coding	0.332	0.303	-0.029	4.173	3.281	0.045	black
ENSG00000081059	TCF7	transcription factor 7 [Source:HGNC Symbol;Acc:HGNC:11639]	protein_coding	0.692	0.382	-0.310	0.683	4.538	0.015	black
ENSG00000103855	CD276	CD276 molecule [Source:HGNC Symbol;Acc:HGNC:19137]	protein_coding	-0.160	-0.335	-0.175	3.597	4.537	0.015	black
ENSG00000112195	TREML2	triggering receptor expressed on myeloid cells like 2 [Source:HGNC Symbol;Acc:HGNC:21092]	protein_coding	0.561	1.663	1.102	-2.836	6.854	0.002	black
ENSG00000115607	IL18RAP	interleukin 18 receptor accessory protein [Source:HGNC Symbol;Acc:HGNC:5989]	protein_coding	1.019	1.721	0.702	-1.977	6.032	0.004	black
ENSG00000128383	APOBEC3A	apolipoprotein B mRNA editing enzyme catalytic subunit 3A [Source:HGNC Symbol;Acc:HGNC:17343]	protein_coding	0.719	1.443	0.724	-2.212	3.213	0.048	black
ENSG00000134909	ARHGAP32	Rho GTPase activating protein 32 [Source:HGNC Symbol;Acc:HGNC:17399]	protein_coding	-0.330	-0.274	0.055	2.964	3.300	0.044	black
ENSG00000151789	ZNF385D	zinc finger protein 385D [Source:HGNC Symbol;Acc:HGNC:26191]	protein_coding	-0.849	-0.948	-0.099	1.222	5.439	0.007	black
ENSG00000151948	GLT1D1	glycosyltransferase 1 domain containing 1 [Source:HGNC Symbol;Acc:HGNC:26483]	protein_coding	0.500	1.431	0.931	-2.593	3.845	0.027	black
ENSG00000157551	KCNJ15	potassium inwardly rectifying channel subfamily J member 15 [Source:HGNC Symbol;Acc:HGNC:6261]	protein_coding	0.200	1.144	0.945	-0.392	3.709	0.031	black
ENSG00000158517	NCF1	neutrophil cytosolic factor 1 [Source:HGNC Symbol;Acc:HGNC:7660]	protein_coding	0.444	0.984	0.540	0.283	3.298	0.045	black
ENSG00000162676	GFI1	growth factor independent 1 transcriptional repressor [Source:HGNC Symbol;Acc:HGNC:4237]	protein_coding	1.015	0.148	-0.866	-1.992	3.888	0.026	black
ENSG00000186529	CYP4F3	cytochrome P450 family 4 subfamily F member 3 [Source:HGNC Symbol;Acc:HGNC:2646]	protein_coding	0.466	1.604	1.137	-1.468	3.947	0.025	black
ENSG00000231259	ANAPC1P2	ANAPC1 pseudogene 2 [Source:HGNC Symbol;Acc:HGNC:54708]	unprocessed_pseudogene	0.406	2.080	1.674	-3.395	3.707	0.031	black
ENSG00000280132	AC026471.6	novel transcript	TEC	-0.819	-0.471	0.347	-2.296	3.240	0.047	black
ENSG00000005187	ACSM3	acyl-CoA synthetase medium chain family member 3 [Source:HGNC Symbol;Acc:HGNC:10522]	protein_coding	0.390	0.550	0.159	2.505	3.933	0.025	blue
ENSG00000007908	SELE	selectin E [Source:HGNC Symbol;Acc:HGNC:10718]	protein_coding	-0.227	1.951	2.178	0.162	4.623	0.014	blue
ENSG00000008300	CELSR3	cadherin EGF LAG seven-pass G-type receptor 3 [Source:HGNC Symbol;Acc:HGNC:3230]	protein_coding	0.549	1.329	0.781	-1.310	3.517	0.037	blue

ENSG00000011304	PTBP1	polypyrimidine tract binding protein 1 [Source:HGNC Symbol;Acc:HGNC:9583]	protein_coding	0.003	0.332	0.330	4.042	3.188	0.049	blue
ENSG00000050327	ARHGEF5	Rho guanine nucleotide exchange factor 5 [Source:HGNC Symbol;Acc:HGNC:13209]	protein_coding	0.132	-0.309	-0.441	1.745	3.362	0.042	blue
ENSG00000054282	SDCCAG8	serologically defined colon cancer antigen 8 [Source:HGNC Symbol;Acc:HGNC:10671]	protein_coding	-0.209	-0.057	0.151	3.703	3.171	0.050	blue
ENSG00000069399	BCL3	BCL3 transcription coactivator [Source:HGNC Symbol;Acc:HGNC:998]	protein_coding	-0.387	0.194	0.581	1.358	3.928	0.026	blue
ENSG00000072182	ASIC4	acid sensing ion channel subunit family member 4 [Source:HGNC Symbol;Acc:HGNC:21263]	protein_coding	1.217	0.334	-0.883	-2.353	3.435	0.039	blue
ENSG00000081148	IMPG2	interphotoreceptor matrix proteoglycan 2 [Source:HGNC Symbol;Acc:HGNC:18362]	protein_coding	-0.299	0.132	0.431	-0.159	3.237	0.047	blue
ENSG00000087074	PPP1R15A	protein phosphatase 1 regulatory subunit 15A [Source:HGNC Symbol;Acc:HGNC:14375]	protein_coding	-0.183	0.259	0.442	5.265	4.776	0.012	blue
ENSG00000099860	GADD45B	growth arrest and DNA damage inducible beta [Source:HGNC Symbol;Acc:HGNC:4096]	protein_coding	-0.279	0.422	0.701	4.460	3.543	0.036	blue
ENSG00000100450	GZMH	granzyme H [Source:HGNC Symbol;Acc:HGNC:4710]	protein_coding	0.795	0.374	-0.421	-1.574	3.418	0.040	blue
ENSG00000102390	PBDC1	polysaccharide biosynthesis domain containing 1 [Source:HGNC Symbol;Acc:HGNC:28790]	protein_coding	-0.313	-0.327	-0.014	0.752	3.293	0.045	blue
ENSG00000104368	PLAT	plasminogen activator, tissue type [Source:HGNC Symbol;Acc:HGNC:9051]	protein_coding	-0.341	0.103	0.444	3.573	3.830	0.028	blue
ENSG00000104490	NCALD	neurocalcin delta [Source:HGNC Symbol;Acc:HGNC:7655]	protein_coding	-0.247	0.101	0.349	3.042	3.462	0.039	blue
ENSG00000105835	NAMPT	nicotinamide phosphoribosyltransferase [Source:HGNC Symbol;Acc:HGNC:30092]	protein_coding	-0.232	0.135	0.366	6.281	3.584	0.035	blue
ENSG00000108342	CSF3	colony stimulating factor 3 [Source:HGNC Symbol;Acc:HGNC:2438]	protein_coding	0.090	1.546	1.457	-3.534	3.477	0.038	blue
ENSG00000108691	CCL2	C-C motif chemokine ligand 2 [Source:HGNC Symbol;Acc:HGNC:10618]	protein_coding	-0.557	0.424	0.981	4.125	3.413	0.040	blue
ENSG00000109610	SOD3	superoxide dismutase 3 [Source:HGNC Symbol;Acc:HGNC:11181]	protein_coding	0.242	0.033	-0.210	3.881	4.076	0.022	blue
ENSG00000111424	VDR	vitamin D receptor [Source:HGNC Symbol;Acc:HGNC:12679]	protein_coding	-0.886	-0.412	0.474	-1.088	3.682	0.032	blue
ENSG00000111912	NCOA7	nuclear receptor coactivator 7 [Source:HGNC Symbol;Acc:HGNC:21081]	protein_coding	-0.350	0.208	0.558	3.768	4.516	0.015	blue
ENSG00000113356	POLR3G	RNA polymerase III subunit G [Source:HGNC Symbol;Acc:HGNC:30075]	protein_coding	-0.699	0.107	0.806	-0.682	4.681	0.013	blue
ENSG00000115665	SLC5A7	solute carrier family 5 member 7 [Source:HGNC Symbol;Acc:HGNC:14025]	protein_coding	0.759	0.382	-0.377	-0.730	3.882	0.027	blue
ENSG00000117036	ETV3	ETS variant transcription factor 3 [Source:HGNC Symbol;Acc:HGNC:3492]	protein_coding	-0.171	0.017	0.188	3.442	3.613	0.034	blue
ENSG00000117479	SLC19A2	solute carrier family 19 member 2 [Source:HGNC Symbol;Acc:HGNC:10938]	protein_coding	-0.328	0.112	0.441	3.129	3.694	0.031	blue

ENSG00000119231	SENP5	SUMO specific peptidase 5 [Source:HGNC Symbol;Acc:HGNC:28407]	protein_coding	-0.236	-0.054	0.182	4.128	4.827	0.012	blue
ENSG00000121101	TEX14	testis expressed 14, intercellular bridge forming factor [Source:HGNC Symbol;Acc:HGNC:11737]	protein_coding	-0.623	0.621	1.244	-2.762	3.489	0.038	blue
ENSG00000124831	LRRFIP1	LRR binding FLII interacting protein 1 [Source:HGNC Symbol;Acc:HGNC:6702]	protein_coding	0.121	0.282	0.161	5.483	5.830	0.005	blue
ENSG00000125148	MT2A	metallothionein 2A [Source:HGNC Symbol;Acc:HGNC:7406]	protein_coding	-0.267	0.262	0.529	3.039	3.349	0.043	blue
ENSG00000131873	CHSY1	chondroitin sulfate synthase 1 [Source:HGNC Symbol;Acc:HGNC:17198]	protein_coding	-0.113	0.222	0.335	4.054	3.919	0.026	blue
ENSG00000132002	DNAJB1	DnaJ heat shock protein family (Hsp40) member B1 [Source:HGNC Symbol;Acc:HGNC:5270]	protein_coding	-0.152	0.106	0.258	4.141	3.444	0.039	blue
ENSG00000132680	KHDC4	KH domain containing 4, pre-mRNA splicing factor [Source:HGNC Symbol;Acc:HGNC:29145]	protein_coding	0.187	0.072	-0.115	3.430	3.224	0.048	blue
ENSG00000133874	RNF122	ring finger protein 122 [Source:HGNC Symbol;Acc:HGNC:21147]	protein_coding	-0.335	0.163	0.498	1.842	4.665	0.014	blue
ENSG00000136011	STAB2	stabilin 2 [Source:HGNC Symbol;Acc:HGNC:18629]	protein_coding	0.746	1.809	1.063	0.113	5.282	0.008	blue
ENSG00000138166	DUSP5	dual specificity phosphatase 5 [Source:HGNC Symbol;Acc:HGNC:3071]	protein_coding	-0.192	0.635	0.827	1.719	4.758	0.013	blue
ENSG00000140379	BCL2A1	BCL2 related protein A1 [Source:HGNC Symbol;Acc:HGNC:991]	protein_coding	-0.450	0.792	1.243	-1.444	4.978	0.010	blue
ENSG00000140406	TLNRD1	talin rod domain containing 1 [Source:HGNC Symbol;Acc:HGNC:13519]	protein_coding	-0.320	-0.037	0.283	2.440	3.270	0.046	blue
ENSG00000141698	NT5C3B	5'-nucleotidase, cytosolic IIIB [Source:HGNC Symbol;Acc:HGNC:28300]	protein_coding	-0.205	-0.260	-0.054	2.528	3.594	0.034	blue
ENSG00000143320	CRABP2	cellular retinoic acid binding protein 2 [Source:HGNC Symbol;Acc:HGNC:2339]	protein_coding	-0.678	0.122	0.801	-1.537	3.293	0.045	blue
ENSG00000157557	ETS2	ETS proto-oncogene 2, transcription factor [Source:HGNC Symbol;Acc:HGNC:3489]	protein_coding	-0.146	0.181	0.327	4.453	4.183	0.020	blue
ENSG00000159167	STC1	stanniocalcin 1 [Source:HGNC Symbol;Acc:HGNC:11373]	protein_coding	0.045	0.783	0.737	1.819	5.021	0.010	blue
ENSG00000159339	PADI4	peptidyl arginine deiminase 4 [Source:HGNC Symbol;Acc:HGNC:18368]	protein_coding	0.310	1.329	1.019	-2.660	3.572	0.035	blue
ENSG00000160785	SLC25A44	solute carrier family 25 member 44 [Source:HGNC Symbol;Acc:HGNC:29036]	protein_coding	-0.184	0.056	0.240	3.205	4.932	0.011	blue
ENSG00000163159	VPS72	vacuolar protein sorting 72 homolog [Source:HGNC Symbol;Acc:HGNC:11644]	protein_coding	0.102	-0.107	-0.209	2.964	3.568	0.035	blue
ENSG00000163638	ADAMTS9	ADAM metallopeptidase with thrombospondin type 1 motif 9 [Source:HGNC Symbol;Acc:HGNC:13202]	protein_coding	0.077	0.746	0.669	2.790	6.067	0.004	blue
ENSG00000163659	TIPARP	TCDD inducible poly(ADP-ribose) polymerase [Source:HGNC Symbol;Acc:HGNC:23696]	protein_coding	-0.233	0.131	0.364	4.737	5.080	0.010	blue
ENSG00000163909	HEYL	hes related family bHLH transcription factor with YRPW motif like [Source:HGNC Symbol;Acc:HGNC:4882]	protein_coding	0.090	0.279	0.188	4.254	3.662	0.032	blue

ENSG00000164120	HPGD	15-hydroxyprostaglandin dehydrogenase [Source:HGNC Symbol;Acc:HGNC:5154]	protein_coding	-0.789	-0.153	0.636	-0.951	6.485	0.003	blue
ENSG00000164949	GEM	GTP binding protein overexpressed in skeletal muscle [Source:HGNC Symbol;Acc:HGNC:4234]	protein_coding	-0.325	0.352	0.678	3.652	3.528	0.036	blue
ENSG00000165030	NFIL3	nuclear factor, interleukin 3 regulated [Source:HGNC Symbol;Acc:HGNC:7787]	protein_coding	-0.308	0.196	0.504	3.114	4.548	0.015	blue
ENSG00000171236	LRG1	leucine rich alpha-2-glycoprotein 1 [Source:HGNC Symbol;Acc:HGNC:29480]	protein_coding	-0.327	1.237	1.564	-2.058	6.716	0.002	blue
ENSG00000171617	ENC1	ectodermal-neural cortex 1 [Source:HGNC Symbol;Acc:HGNC:3345]	protein_coding	-0.311	0.080	0.391	2.751	3.936	0.025	blue
ENSG00000172530	BANP	BTG3 associated nuclear protein [Source:HGNC Symbol;Acc:HGNC:13450]	protein_coding	-0.187	0.272	0.459	2.138	3.810	0.028	blue
ENSG00000172602	RND1	Rho family GTPase 1 [Source:HGNC Symbol;Acc:HGNC:18314]	protein_coding	-0.032	1.742	1.774	-1.368	6.852	0.002	blue
ENSG00000172831	CES2	carboxylesterase 2 [Source:HGNC Symbol;Acc:HGNC:1864]	protein_coding	0.136	-0.023	-0.159	4.210	3.635	0.033	blue
ENSG00000173530	TNFRSF10D	TNF receptor superfamily member 10d [Source:HGNC Symbol;Acc:HGNC:11907]	protein_coding	-0.141	0.364	0.505	1.411	3.238	0.047	blue
ENSG00000178726	THBD	thrombomodulin [Source:HGNC Symbol;Acc:HGNC:11784]	protein_coding	-0.259	0.304	0.563	3.371	4.473	0.016	blue
ENSG00000179082	C9orf106	chromosome 9 putative open reading frame 106 [Source:HGNC Symbol;Acc:HGNC:31370]	lncRNA	0.727	-0.628	-1.355	-1.877	7.572	0.001	blue
ENSG00000180596	H2BC4	H2B clustered histone 4 [Source:HGNC Symbol;Acc:HGNC:4757]	protein_coding	0.177	-0.028	-0.205	3.297	3.405	0.041	blue
ENSG00000184588	PDE4B	phosphodiesterase 4B [Source:HGNC Symbol;Acc:HGNC:8781]	protein_coding	-0.363	0.044	0.407	4.575	7.698	0.001	blue
ENSG00000185022	MAFF	MAF bZIP transcription factor F [Source:HGNC Symbol;Acc:HGNC:6780]	protein_coding	-0.378	0.535	0.913	2.439	3.421	0.040	blue
ENSG00000186056	MATN1-AS1	MATN1 antisense RNA 1 [Source:HGNC Symbol;Acc:HGNC:40364]	lncRNA	0.519	-0.749	-1.269	-1.509	4.168	0.021	blue
ENSG00000186407	CD300E	CD300e molecule [Source:HGNC Symbol;Acc:HGNC:28874]	protein_coding	0.095	1.070	0.975	-0.810	5.256	0.008	blue
ENSG00000187140	FOXD3	forkhead box D3 [Source:HGNC Symbol;Acc:HGNC:3804]	protein_coding	0.345	-0.251	-0.597	-0.484	3.331	0.043	blue
ENSG00000187624	C17orf97	chromosome 17 open reading frame 97 [Source:HGNC Symbol;Acc:HGNC:33800]	protein_coding	0.659	-0.078	-0.737	0.250	3.295	0.045	blue
ENSG00000196189	SEMA4A	semaphorin 4A [Source:HGNC Symbol;Acc:HGNC:10729]	protein_coding	-0.780	0.596	1.377	1.266	4.446	0.016	blue
ENSG00000196843	ARID5A	AT-rich interaction domain 5A [Source:HGNC Symbol;Acc:HGNC:17361]	protein_coding	-0.103	0.435	0.537	2.619	4.311	0.018	blue
ENSG00000197063	MAFG	MAF bZIP transcription factor G [Source:HGNC Symbol;Acc:HGNC:6781]	protein_coding	0.006	0.262	0.256	3.370	3.276	0.045	blue
ENSG00000203392	AC105020.1	novel transcript, antisense to CSPG4	lncRNA	-0.667	-0.224	0.443	-2.638	3.552	0.036	blue

ENSG00000205362	MT1A	metallothionein 1A [Source:HGNC Symbol;Acc:HGNC:7393]	protein_coding	-0.536	1.389	1.925	-1.896	7.349	0.002	blue
ENSG00000205502	C2CD4B	C2 calcium dependent domain containing 4B [Source:HGNC Symbol;Acc:HGNC:33628]	protein_coding	-0.145	1.343	1.488	-1.386	4.024	0.024	blue
ENSG00000205710	C17orf107	chromosome 17 open reading frame 107 [Source:HGNC Symbol;Acc:HGNC:37238]	protein_coding	-0.507	0.031	0.539	0.243	3.571	0.035	blue
ENSG00000213753	CENPBD1P1	CENPB DNA-binding domains containing 1 pseudogene 1 [Source:HGNC Symbol;Acc:HGNC:28421]	transcribed_processed_pseudogene	-0.063	0.476	0.539	3.360	4.367	0.017	blue
ENSG00000215022	AL008729.1	novel transcript, antisense to PHACTR1	lncRNA	-1.104	-0.510	0.594	-2.011	4.738	0.013	blue
ENSG00000218336	TENM3	teneurin transmembrane protein 3 [Source:HGNC Symbol;Acc:HGNC:29944]	protein_coding	0.206	-0.310	-0.516	3.341	4.249	0.019	blue
ENSG00000229828	PDE4DIPP1	phosphodiesterase 4D interacting protein pseudogene 1 [Source:HGNC Symbol;Acc:HGNC:50867]	unprocessed_pseudogene	1.137	-1.068	-2.205	-3.373	3.685	0.032	blue
ENSG00000229927	RHEBP1	RHEB pseudogene 1 [Source:HGNC Symbol;Acc:HGNC:10010]	processed_pseudogene	1.055	-0.912	-1.967	-2.919	3.470	0.038	blue
ENSG00000230658	KLHL7-DT	KLHL7 divergent transcript [Source:HGNC Symbol;Acc:HGNC:43431]	lncRNA	-1.168	-0.020	1.148	-1.819	6.573	0.003	blue
ENSG00000237513	AC007384.1	novel transcript	lncRNA	-0.207	0.783	0.990	-1.425	3.677	0.032	blue
ENSG00000237928	NFIA-AS2	NFIA antisense RNA 2 [Source:HGNC Symbol;Acc:HGNC:40401]	lncRNA	-0.712	-0.599	0.114	-0.666	3.829	0.028	blue
ENSG00000239713	APOBEC3G	apolipoprotein B mRNA editing enzyme catalytic subunit 3G [Source:HGNC Symbol;Acc:HGNC:17357]	protein_coding	0.310	0.117	-0.192	1.534	4.143	0.021	blue
ENSG00000249839	AC011330.1	histidine acid phosphatase domain containing 2A (HISPPD2A) pseudogene	unprocessed_pseudogene	2.365	0.372	-1.993	-2.799	5.169	0.009	blue
ENSG00000250312	ZNF718	zinc finger protein 718 [Source:HGNC Symbol;Acc:HGNC:26889]	protein_coding	0.171	-0.399	-0.571	1.365	3.867	0.027	blue
ENSG00000250506	CDK3	cyclin dependent kinase 3 [Source:HGNC Symbol;Acc:HGNC:1772]	protein_coding	-0.909	0.250	1.160	-1.755	3.429	0.040	blue
ENSG00000251441	RTEL1P1	regulator of telomere elongation helicase 1 pseudogene 1 [Source:HGNC Symbol;Acc:HGNC:44213]	transcribed_processed_pseudogene	0.660	-0.368	-1.028	-2.497	3.763	0.030	blue
ENSG00000255717	SNHG1	small nucleolar RNA host gene 1 [Source:HGNC Symbol;Acc:HGNC:32688]	lncRNA	0.076	-0.339	-0.415	1.948	4.936	0.011	blue
ENSG00000255819	KLRC4-KLRK1	KLRC4-KLRK1 readthrough [Source:HGNC Symbol;Acc:HGNC:48357]	protein_coding	1.177	-0.077	-1.253	-2.951	3.482	0.038	blue
ENSG00000256463	SALL3	spalt like transcription factor 3 [Source:HGNC Symbol;Acc:HGNC:10527]	protein_coding	0.102	0.880	0.778	-0.631	3.320	0.044	blue
ENSG00000272446	AL158850.1	novel transcript	lncRNA	0.476	-0.085	-0.561	-0.609	4.162	0.021	blue
ENSG00000272617	AC026464.6	novel protein, COG8-PDF readthrough	protein_coding	1.371	-0.809	-2.180	-3.284	6.696	0.003	blue
ENSG00000277283	AC004812.2	novel transcript, antisense to RAB35	lncRNA	0.078	-0.631	-0.709	-0.690	4.947	0.011	blue

ENSG00000277363	SRCIN1	SRC kinase signaling inhibitor 1 [Source:HGNC Symbol;Acc:HGNC:29506]	protein_coding	-0.279	0.435	0.714	0.321	3.882	0.027	blue
ENSG00000278989	AP001148.1	novel transcript	TEC	0.314	-0.506	-0.820	-1.567	3.457	0.039	blue
ENSG00000280077	AL353763.2	TEC	TEC	-0.094	-0.527	-0.433	-0.016	3.364	0.042	blue
ENSG00000280594	BTG3-AS1	BTG3 antisense RNA 1 [Source:HGNC Symbol;Acc:HGNC:53145]	lncRNA	-0.354	0.847	1.201	-2.447	6.519	0.003	blue
ENSG00000285679	AC079142.1	novel transcript	lncRNA	-0.088	-0.559	-0.470	-1.239	3.201	0.049	blue
ENSG00000286235	AL035461.4	novel protein	protein_coding	1.801	-0.874	-2.676	-3.893	4.982	0.010	blue
ENSG00000288253	AC010332.3	novel transcript	lncRNA	0.691	-0.918	-1.610	-2.907	3.933	0.025	blue
ENSG00000288534	AP001931.2	TMX2-CTNND1 readthrough (NMD candidate)	protein_coding	-1.012	-0.084	0.929	3.431	3.623	0.033	blue
ENSG00000019549	SNAI2	snail family transcriptional repressor 2 [Source:HGNC Symbol;Acc:HGNC:11094]	protein_coding	-0.149	-0.417	-0.268	2.793	3.550	0.036	brown
ENSG00000036257	CUL3	cullin 3 [Source:HGNC Symbol;Acc:HGNC:2553]	protein_coding	0.237	0.158	-0.079	4.597	5.665	0.006	brown
ENSG00000049449	RCN1	reticulocalbin 1 [Source:HGNC Symbol;Acc:HGNC:9934]	protein_coding	-0.268	-0.486	-0.218	3.853	5.442	0.007	brown
ENSG00000070526	ST6GALNAC1	ST6 N-acetylgalactosaminide alpha-2,6-sialyltransferase 1 [Source:HGNC Symbol;Acc:HGNC:23614]	protein_coding	0.355	0.755	0.399	0.172	4.040	0.023	brown
ENSG00000100664	EIF5	eukaryotic translation initiation factor 5 [Source:HGNC Symbol;Acc:HGNC:3299]	protein_coding	0.202	0.157	-0.045	6.467	6.477	0.003	brown
ENSG00000101191	DIDO1	death inducer-obliterator 1 [Source:HGNC Symbol;Acc:HGNC:2680]	protein_coding	0.095	0.136	0.042	4.853	4.001	0.024	brown
ENSG00000101413	RPRD1B	regulation of nuclear pre-mRNA domain containing 1B [Source:HGNC Symbol;Acc:HGNC:16209]	protein_coding	0.210	0.374	0.164	4.496	4.164	0.021	brown
ENSG00000101938	CHRDL1	chordin like 1 [Source:HGNC Symbol;Acc:HGNC:29861]	protein_coding	-0.271	-0.525	-0.255	4.534	3.324	0.044	brown
ENSG00000104313	EYA1	EYA transcriptional coactivator and phosphatase 1 [Source:HGNC Symbol;Acc:HGNC:3519]	protein_coding	0.467	0.743	0.276	2.425	5.518	0.007	brown
ENSG00000105499	PLA2G4C	phospholipase A2 group IVC [Source:HGNC Symbol;Acc:HGNC:9037]	protein_coding	0.413	0.493	0.080	3.074	4.491	0.016	brown
ENSG00000108239	TBC1D12	TBC1 domain family member 12 [Source:HGNC Symbol;Acc:HGNC:29082]	protein_coding	-0.220	-0.204	0.017	3.026	3.612	0.034	brown
ENSG00000110881	ASIC1	acid sensing ion channel subunit 1 [Source:HGNC Symbol;Acc:HGNC:100]	protein_coding	0.008	-0.582	-0.589	-0.140	3.196	0.049	brown
ENSG00000111224	PARP11	poly(ADP-ribose) polymerase family member 11 [Source:HGNC Symbol;Acc:HGNC:1186]	protein_coding	-0.503	-0.422	0.080	1.232	3.292	0.045	brown
ENSG00000116774	OLFML3	olfactomedin like 3 [Source:HGNC Symbol;Acc:HGNC:24956]	protein_coding	-0.343	-0.463	-0.121	4.225	3.705	0.031	brown

ENSG00000117020	AKT3	AKT serine/threonine kinase 3 [Source:HGNC Symbol;Acc:HGNC:393]	protein_coding	-0.222	-0.167	0.055	5.009	4.142	0.021	brown
ENSG00000117868	ESYT2	extended synaptotagmin 2 [Source:HGNC Symbol;Acc:HGNC:22211]	protein_coding	-0.178	-0.130	0.048	5.883	4.313	0.018	brown
ENSG00000118194	TNNT2	troponin T2, cardiac type [Source:HGNC Symbol;Acc:HGNC:11949]	protein_coding	0.126	0.378	0.253	10.245	4.575	0.015	brown
ENSG00000119682	AREL1	apoptosis resistant E3 ubiquitin protein ligase 1 [Source:HGNC Symbol;Acc:HGNC:20363]	protein_coding	0.267	0.462	0.195	3.489	4.362	0.018	brown
ENSG00000120820	GLT8D2	glycosyltransferase 8 domain containing 2 [Source:HGNC Symbol;Acc:HGNC:24890]	protein_coding	-0.397	-0.417	-0.020	2.425	4.313	0.018	brown
ENSG00000120910	PPP3CC	protein phosphatase 3 catalytic subunit gamma [Source:HGNC Symbol;Acc:HGNC:9316]	protein_coding	-0.086	0.223	0.309	3.423	3.409	0.040	brown
ENSG00000122707	RECK	reversion inducing cysteine rich protein with kazal motifs [Source:HGNC Symbol;Acc:HGNC:11345]	protein_coding	-0.258	-0.252	0.006	3.082	3.271	0.046	brown
ENSG00000122870	BICC1	BicC family RNA binding protein 1 [Source:HGNC Symbol;Acc:HGNC:19351]	protein_coding	-0.286	-0.414	-0.128	4.523	4.033	0.023	brown
ENSG00000129226	CD68	CD68 molecule [Source:HGNC Symbol;Acc:HGNC:1693]	protein_coding	-0.299	-0.586	-0.287	4.540	4.210	0.020	brown
ENSG00000132780	NASP	nuclear autoantigenic sperm protein [Source:HGNC Symbol;Acc:HGNC:7644]	protein_coding	0.167	0.251	0.083	3.843	3.803	0.029	brown
ENSG00000134917	ADAMTS8	ADAM metallopeptidase with thrombospondin type 1 motif 8 [Source:HGNC Symbol;Acc:HGNC:224]	protein_coding	-0.660	-0.890	-0.230	-0.940	5.345	0.008	brown
ENSG00000136999	CCN3	cellular communication network factor 3 [Source:HGNC Symbol;Acc:HGNC:7885]	protein_coding	-0.421	-0.178	0.243	2.767	3.434	0.039	brown
ENSG00000140092	FBLN5	fibulin 5 [Source:HGNC Symbol;Acc:HGNC:3602]	protein_coding	-0.261	-0.393	-0.132	5.737	3.873	0.027	brown
ENSG00000140254	DUOXA1	dual oxidase maturation factor 1 [Source:HGNC Symbol;Acc:HGNC:26507]	protein_coding	-0.532	-1.044	-0.512	-1.056	3.827	0.028	brown
ENSG00000141232	TOB1	transducer of ERBB2, 1 [Source:HGNC Symbol;Acc:HGNC:11979]	protein_coding	-0.228	-0.251	-0.023	3.660	3.573	0.035	brown
ENSG00000141252	VPS53	VPS53 subunit of GARP complex [Source:HGNC Symbol;Acc:HGNC:25608]	protein_coding	0.012	0.353	0.341	3.760	4.482	0.016	brown
ENSG00000143344	RGL1	ral guanine nucleotide dissociation stimulator like 1 [Source:HGNC Symbol;Acc:HGNC:30281]	protein_coding	-0.096	-0.256	-0.160	4.624	3.322	0.044	brown
ENSG00000145147	SLIT2	slit guidance ligand 2 [Source:HGNC Symbol;Acc:HGNC:11086]	protein_coding	0.072	-0.267	-0.339	3.931	5.314	0.008	brown
ENSG00000147255	IGSF1	immunoglobulin superfamily member 1 [Source:HGNC Symbol;Acc:HGNC:5948]	protein_coding	0.710	0.785	0.075	3.638	3.518	0.037	brown
ENSG00000147394	ZNF185	zinc finger protein 185 with LIM domain [Source:HGNC Symbol;Acc:HGNC:12976]	protein_coding	-0.482	-0.556	-0.073	0.709	3.963	0.025	brown
ENSG00000148082	SHC3	SHC adaptor protein 3 [Source:HGNC Symbol;Acc:HGNC:18181]	protein_coding	-0.794	-0.579	0.215	1.601	5.491	0.007	brown
ENSG00000148344	PTGES	prostaglandin E synthase [Source:HGNC Symbol;Acc:HGNC:9599]	protein_coding	-0.578	-0.782	-0.204	0.217	3.931	0.025	brown

ENSG00000148700	ADD3	adducin 3 [Source:HGNC Symbol;Acc:HGNC:245]	protein_coding	-0.160	-0.228	-0.069	6.483	3.947	0.025	brown
ENSG00000149090	PAMR1	peptidase domain containing associated with muscle regeneration 1 [Source:HGNC Symbol;Acc:HGNC:24554]	protein_coding	-0.812	-0.817	-0.005	3.052	6.267	0.004	brown
ENSG00000153707	PTPRD	protein tyrosine phosphatase receptor type D [Source:HGNC Symbol;Acc:HGNC:9668]	protein_coding	-0.710	-0.574	0.136	2.598	3.432	0.040	brown
ENSG00000154263	ABCA10	ATP binding cassette subfamily A member 10 [Source:HGNC Symbol;Acc:HGNC:30]	protein_coding	0.080	-0.466	-0.545	2.739	3.272	0.046	brown
ENSG00000156097	GPR61	G protein-coupled receptor 61 [Source:HGNC Symbol;Acc:HGNC:13300]	protein_coding	1.073	0.014	-1.059	-2.462	3.250	0.046	brown
ENSG00000156466	GDF6	growth differentiation factor 6 [Source:HGNC Symbol;Acc:HGNC:4221]	protein_coding	-0.449	-0.703	-0.254	1.077	4.000	0.024	brown
ENSG00000162407	PLPP3	phospholipid phosphatase 3 [Source:HGNC Symbol;Acc:HGNC:9229]	protein_coding	-0.227	-0.257	-0.029	5.571	3.784	0.029	brown
ENSG00000162616	DNAJB4	DnaJ heat shock protein family (Hsp40) member B4 [Source:HGNC Symbol;Acc:HGNC:14886]	protein_coding	0.045	0.224	0.179	5.020	3.171	0.050	brown
ENSG00000162733	DDR2	discoidin domain receptor tyrosine kinase 2 [Source:HGNC Symbol;Acc:HGNC:2731]	protein_coding	-0.091	-0.209	-0.118	6.606	4.449	0.016	brown
ENSG00000164591	MYOZ3	myozenin 3 [Source:HGNC Symbol;Acc:HGNC:18565]	protein_coding	0.472	0.616	0.144	1.180	3.789	0.029	brown
ENSG00000165072	MAMDC2	MAM domain containing 2 [Source:HGNC Symbol;Acc:HGNC:23673]	protein_coding	-0.310	-0.152	0.158	3.638	3.734	0.030	brown
ENSG00000166145	SPINT1	serine peptidase inhibitor, Kunitz type 1 [Source:HGNC Symbol;Acc:HGNC:11246]	protein_coding	-0.856	-1.288	-0.432	-0.811	3.971	0.025	brown
ENSG00000166507	NDST2	N-deacetylase and N-sulfotransferase 2 [Source:HGNC Symbol;Acc:HGNC:7681]	protein_coding	-0.144	-0.462	-0.318	2.006	4.399	0.017	brown
ENSG00000166925	TSC22D4	TSC22 domain family member 4 [Source:HGNC Symbol;Acc:HGNC:21696]	protein_coding	0.243	0.118	-0.125	3.740	4.501	0.016	brown
ENSG00000166949	SMAD3	SMAD family member 3 [Source:HGNC Symbol;Acc:HGNC:6769]	protein_coding	-0.166	-0.377	-0.211	4.335	4.658	0.014	brown
ENSG00000169604	ANTXR1	ANTXR cell adhesion molecule 1 [Source:HGNC Symbol;Acc:HGNC:21014]	protein_coding	-0.352	-0.408	-0.057	5.886	6.827	0.002	brown
ENSG00000174348	PODN	podocan [Source:HGNC Symbol;Acc:HGNC:23174]	protein_coding	-0.205	-0.460	-0.255	4.579	6.754	0.002	brown
ENSG00000175198	PCCA	propionyl-CoA carboxylase subunit alpha [Source:HGNC Symbol;Acc:HGNC:8653]	protein_coding	-0.298	-0.226	0.072	3.637	3.840	0.028	brown
ENSG00000176209	SMIM19	small integral membrane protein 19 [Source:HGNC Symbol;Acc:HGNC:25166]	protein_coding	-0.067	0.198	0.265	3.241	3.990	0.024	brown
ENSG00000178028	DMAP1	DNA methyltransferase 1 associated protein 1 [Source:HGNC Symbol;Acc:HGNC:18291]	protein_coding	0.323	0.182	-0.141	2.342	4.047	0.023	brown
ENSG00000180530	NRIP1	nuclear receptor interacting protein 1 [Source:HGNC Symbol;Acc:HGNC:8001]	protein_coding	-0.162	-0.290	-0.128	4.880	4.431	0.017	brown
ENSG00000183117	CSMD1	CUB and Sushi multiple domains 1 [Source:HGNC Symbol;Acc:HGNC:14026]	protein_coding	1.358	3.326	1.968	-2.131	6.799	0.002	brown

ENSG00000184060	ADAP2	ArfGAP with dual PH domains 2 [Source:HGNC Symbol;Acc:HGNC:16487]	protein_coding	-0.273	-0.534	-0.262	2.402	3.471	0.038	brown
ENSG00000186818	LILRB4	leukocyte immunoglobulin like receptor B4 [Source:HGNC Symbol;Acc:HGNC:6608]	protein_coding	-0.387	-0.579	-0.192	0.953	3.549	0.036	brown
ENSG00000187210	GCNT1	glucosaminyl (N-acetyl) transferase 1 [Source:HGNC Symbol;Acc:HGNC:4203]	protein_coding	0.500	-0.476	-0.976	1.811	3.623	0.033	brown
ENSG00000189046	ALKBH2	alkB homolog 2, alpha-ketoglutarate dependent dioxygenase [Source:HGNC Symbol;Acc:HGNC:32487]	protein_coding	0.176	0.427	0.250	0.965	3.254	0.046	brown
ENSG00000196368	NUDT11	nudix hydrolase 11 [Source:HGNC Symbol;Acc:HGNC:18011]	protein_coding	-0.758	-0.844	-0.085	-2.243	3.170	0.050	brown
ENSG00000197021	EOLA2	endothelium and lymphocyte associated ASCH domain 2 [Source:HGNC Symbol;Acc:HGNC:17402]	protein_coding	0.163	0.403	0.240	1.762	3.938	0.025	brown
ENSG00000197766	CFD	complement factor D [Source:HGNC Symbol;Acc:HGNC:2771]	protein_coding	-0.182	-0.679	-0.497	5.791	4.183	0.020	brown
ENSG00000197863	ZNF790	zinc finger protein 790 [Source:HGNC Symbol;Acc:HGNC:33114]	protein_coding	0.179	0.472	0.293	2.315	4.148	0.021	brown
ENSG00000198121	LPAR1	lysophosphatidic acid receptor 1 [Source:HGNC Symbol;Acc:HGNC:3166]	protein_coding	-0.278	-0.328	-0.050	4.301	3.732	0.030	brown
ENSG00000198744	MTCO3P12	MT-CO3 pseudogene 12 [Source:HGNC Symbol;Acc:HGNC:52042]	unprocessed_pseudogene	0.451	2.302	1.850	1.088	6.646	0.003	brown
ENSG00000198863	RUNDC1	RUN domain containing 1 [Source:HGNC Symbol;Acc:HGNC:25418]	protein_coding	0.174	0.305	0.130	2.210	3.819	0.028	brown
ENSG00000205037	AC134312.1	novel transcript	lncRNA	0.355	1.566	1.211	-2.261	4.727	0.013	brown
ENSG00000205336	ADGRG1	adhesion G protein-coupled receptor G1 [Source:HGNC Symbol;Acc:HGNC:4512]	protein_coding	0.720	0.657	-0.063	1.657	5.916	0.005	brown
ENSG00000224043	CCNT2-AS1	CCNT2 antisense RNA 1 [Source:HGNC Symbol;Acc:HGNC:40130]	lncRNA	0.739	0.782	0.042	0.912	7.934	0.001	brown
ENSG00000235655	H3P6	H3 histone pseudogene 6 [Source:HGNC Symbol;Acc:HGNC:42980]	processed_pseudogene	0.112	0.591	0.480	-1.586	5.247	0.008	brown
ENSG00000235823	OLMALINC	oligodendrocyte maturation-associated long intergenic non-coding RNA [Source:HGNC Symbol;Acc:HGNC:28060]	lncRNA	0.323	0.159	-0.164	1.695	3.276	0.045	brown
ENSG00000236824	BCYRN1	brain cytoplasmic RNA 1 [Source:HGNC Symbol;Acc:HGNC:1022]	scRNA	0.427	0.303	-0.124	3.654	3.308	0.044	brown
ENSG00000237596	AL138828.1	novel transcript	lncRNA	0.320	0.949	0.629	1.742	4.358	0.018	brown
ENSG00000245812	LINC02202	long intergenic non-protein coding RNA 2202 [Source:HGNC Symbol;Acc:HGNC:53068]	lncRNA	0.367	-0.608	-0.975	-0.475	4.117	0.022	brown
ENSG00000248333	CDK11B	cyclin dependent kinase 11B [Source:HGNC Symbol;Acc:HGNC:1729]	protein_coding	0.268	0.292	0.023	2.522	5.361	0.008	brown
ENSG00000253710	ALG11	ALG11 alpha-1,2-mannosyltransferase [Source:HGNC Symbol;Acc:HGNC:32456]	protein_coding	0.243	0.146	-0.097	4.173	4.942	0.011	brown
ENSG00000258636	AL121821.2	novel transcript	lncRNA	-0.696	-0.409	0.288	-0.518	3.281	0.045	brown

ENSG00000258818	RNASE4	ribonuclease A family member 4 [Source:HGNC Symbol;Acc:HGNC:10047]	protein_coding	-0.281	-0.446	-0.165	3.339	3.580	0.035	brown
ENSG00000267069	AP005264.1	novel transcript	lncRNA	0.646	0.898	0.252	-1.013	4.787	0.012	brown
ENSG00000267530	LINC01836	long intergenic non-protein coding RNA 1836 [Source:HGNC Symbol;Acc:HGNC:52652]	lncRNA	0.831	1.092	0.261	-2.776	3.318	0.044	brown
ENSG00000269896	AL513477.1	small nuclear ribonucleoprotein N (SNRPN) pseudogene	transcribed_processed_pseudogene	0.114	-1.026	-1.140	-2.327	4.656	0.014	brown
ENSG00000275835	TUBGCP5	tubulin gamma complex associated protein 5 [Source:HGNC Symbol;Acc:HGNC:18600]	protein_coding	0.353	0.178	-0.176	2.950	4.410	0.017	brown
ENSG00000279030	AC007336.3	novel transcript	TEC	-0.764	-0.685	0.079	-2.255	3.764	0.029	brown
ENSG00000284649	AC009093.8	BTG3 associated nuclear protein (BANP) pseudogene	transcribed_unprocessed_pseudogene	0.237	1.730	1.494	-2.906	5.166	0.009	brown
ENSG00000010319	SEMA3G	semaphorin 3G [Source:HGNC Symbol;Acc:HGNC:30400]	protein_coding	0.510	0.578	0.067	2.984	3.657	0.032	green
ENSG00000067208	EVI5	ecotropic viral integration site 5 [Source:HGNC Symbol;Acc:HGNC:3501]	protein_coding	-0.251	-0.104	0.148	4.886	3.865	0.027	green
ENSG00000074582	BCS1L	BCS1 homolog, ubiquinol-cytochrome c reductase complex chaperone [Source:HGNC Symbol;Acc:HGNC:1020]	protein_coding	0.281	0.165	-0.116	2.151	3.212	0.048	green
ENSG00000102924	CBLN1	cerebellin 1 precursor [Source:HGNC Symbol;Acc:HGNC:1543]	protein_coding	0.939	0.796	-0.143	-0.031	5.556	0.006	green
ENSG00000113361	CDH6	cadherin 6 [Source:HGNC Symbol;Acc:HGNC:1765]	protein_coding	0.563	0.792	0.229	2.100	4.809	0.012	green
ENSG00000124785	NRN1	neuritin 1 [Source:HGNC Symbol;Acc:HGNC:17972]	protein_coding	0.392	0.308	-0.084	2.151	3.896	0.026	green
ENSG00000125851	PCSK2	proprotein convertase subtilisin/kexin type 2 [Source:HGNC Symbol;Acc:HGNC:8744]	protein_coding	0.720	0.163	-0.558	1.669	6.609	0.003	green
ENSG00000125871	MGME1	mitochondrial genome maintenance exonuclease 1 [Source:HGNC Symbol;Acc:HGNC:16205]	protein_coding	0.309	0.399	0.090	1.779	3.214	0.048	green
ENSG00000134460	IL2RA	interleukin 2 receptor subunit alpha [Source:HGNC Symbol;Acc:HGNC:6008]	protein_coding	-1.035	-0.571	0.464	-0.255	4.196	0.020	green
ENSG00000142748	FCN3	ficolin 3 [Source:HGNC Symbol;Acc:HGNC:3625]	protein_coding	1.025	0.696	-0.329	-1.667	4.038	0.023	green
ENSG00000143248	RGS5	regulator of G protein signaling 5 [Source:HGNC Symbol;Acc:HGNC:10001]	protein_coding	0.467	0.313	-0.154	7.130	5.585	0.006	green
ENSG00000144057	ST6GAL2	ST6 beta-galactoside alpha-2,6-sialyltransferase 2 [Source:HGNC Symbol;Acc:HGNC:10861]	protein_coding	0.759	-0.586	-1.346	0.271	6.993	0.002	green
ENSG00000165810	BTNL9	butyrophilin like 9 [Source:HGNC Symbol;Acc:HGNC:24176]	protein_coding	0.654	0.657	0.003	2.582	3.258	0.046	green
ENSG00000168874	ATOH8	atonal bHLH transcription factor 8 [Source:HGNC Symbol;Acc:HGNC:24126]	protein_coding	0.171	-0.024	-0.196	4.236	3.422	0.040	green
ENSG00000177042	TMEM80	transmembrane protein 80 [Source:HGNC Symbol;Acc:HGNC:27453]	protein_coding	0.374	0.666	0.292	1.412	4.596	0.014	green

ENSG00000183153	GJD3	gap junction protein delta 3 [Source:HGNC Symbol;Acc:HGNC:19147]	protein_coding	0.517	0.313	-0.204	0.332	3.644	0.033	green
ENSG00000198597	ZNF536	zinc finger protein 536 [Source:HGNC Symbol;Acc:HGNC:29025]	protein_coding	0.450	0.045	-0.405	1.545	3.998	0.024	green
ENSG00000198712	MT-CO2	mitochondrially encoded cytochrome c oxidase II [Source:HGNC Symbol;Acc:HGNC:7421]	protein_coding	0.208	0.309	0.101	10.360	3.193	0.049	green
ENSG00000205863	C1QTNF9B	C1q and TNF related 9B [Source:HGNC Symbol;Acc:HGNC:34072]	protein_coding	0.112	1.365	1.253	-3.033	3.837	0.028	green
ENSG00000258819	LINC02289	long intergenic non-protein coding RNA 2289 [Source:HGNC Symbol;Acc:HGNC:53205]	lncRNA	0.735	0.637	-0.098	-1.247	3.813	0.028	green
ENSG00000275385	CCL18	C-C motif chemokine ligand 18 [Source:HGNC Symbol;Acc:HGNC:10616]	protein_coding	-1.550	-0.627	0.923	-1.864	3.781	0.029	green
ENSG00000279249	AC007614.1	novel transcript, antisense to CBLN1	lncRNA	0.822	-0.104	-0.927	-2.156	4.502	0.016	green
ENSG00000158578	ALAS2	5'-aminolevulinate synthase 2 [Source:HGNC Symbol;Acc:HGNC:397]	protein_coding	1.281	1.069	-0.212	-0.792	3.639	0.033	greenyellow
ENSG00000188536	HBA2	hemoglobin subunit alpha 2 [Source:HGNC Symbol;Acc:HGNC:4824]	protein_coding	0.663	1.364	0.700	5.305	4.672	0.013	greenyellow
ENSG00000206172	HBA1	hemoglobin subunit alpha 1 [Source:HGNC Symbol;Acc:HGNC:4823]	protein_coding	0.594	1.228	0.634	5.314	3.665	0.032	greenyellow
ENSG00000215559	ANKRD20A11P	ankyrin repeat domain 20 family member A11, pseudogene [Source:HGNC Symbol;Acc:HGNC:42024]	transcribed_unprocessed_pseudogene	-1.534	-0.955	0.578	-2.853	4.308	0.018	greenyellow
ENSG00000232573	RPL3P4	ribosomal protein L3 pseudogene 4 [Source:HGNC Symbol;Acc:HGNC:19805]	processed_pseudogene	1.997	0.091	-1.906	-2.570	5.055	0.010	greenyellow
ENSG00000254692	AL136295.1	novel protein	protein_coding	2.016	2.837	0.821	-2.450	4.636	0.014	greenyellow
ENSG00000280571	AC006059.2	novel protein	protein_coding	0.218	-3.698	-3.916	0.132	18.007	0.000	greenyellow
ENSG00000054179	ENTPD2	ectonucleoside triphosphate diphosphohydrolase 2 [Source:HGNC Symbol;Acc:HGNC:3364]	protein_coding	0.994	0.405	-0.590	-2.448	4.967	0.010	grey
ENSG00000121060	TRIM25	tripartite motif containing 25 [Source:HGNC Symbol;Acc:HGNC:12932]	protein_coding	0.306	0.434	0.128	3.401	3.250	0.047	grey
ENSG00000154040	CABYR	calcium binding tyrosine phosphorylation regulated [Source:HGNC Symbol;Acc:HGNC:15569]	protein_coding	-1.001	-0.324	0.677	-2.742	4.271	0.019	grey
ENSG00000164308	ERAP2	endoplasmic reticulum aminopeptidase 2 [Source:HGNC Symbol;Acc:HGNC:29499]	protein_coding	0.682	-0.547	-1.229	3.103	3.533	0.036	grey
ENSG00000166140	ZFYVE19	zinc finger FYVE-type containing 19 [Source:HGNC Symbol;Acc:HGNC:20758]	protein_coding	-0.197	0.118	0.316	1.987	3.646	0.033	grey

ENSG00000228252	COL6A4P2	collagen type VI alpha 4 pseudogene 2 [Source:HGNC Symbol;Acc:HGNC:38501]	transcribed_unitary_pseudogene	0.843	-0.214	-1.056	-2.876	3.181	0.049	grey
ENSG00000235535	TRDN-AS1	TRDN antisense RNA 1 [Source:HGNC Symbol;Acc:HGNC:40592]	lncRNA	-0.590	0.023	0.612	2.529	4.122	0.022	grey
ENSG00000261175	LINC02188	long intergenic non-protein coding RNA 2188 [Source:HGNC Symbol;Acc:HGNC:53050]	lncRNA	-0.645	0.615	1.260	-2.862	4.171	0.021	grey
ENSG00000266993	AL050343.2	novel transcript, antisense to NRD1	lncRNA	-0.143	-1.296	-1.153	-2.139	4.207	0.020	grey
ENSG00000283809	AC007326.4	novel protein	protein_coding	-1.155	-0.231	0.924	-1.985	3.292	0.045	grey
ENSG00000110880	CORO1C	coronin 1C [Source:HGNC Symbol;Acc:HGNC:2254]	protein_coding	-0.206	-0.193	0.012	4.413	3.885	0.027	magenta
ENSG00000113966	ARL6	ADP ribosylation factor like GTPase 6 [Source:HGNC Symbol;Acc:HGNC:13210]	protein_coding	-0.282	-0.463	-0.182	1.603	3.169	0.050	magenta
ENSG00000142856	ITGB3BP	integrin subunit beta 3 binding protein [Source:HGNC Symbol;Acc:HGNC:6157]	protein_coding	-0.698	-0.577	0.121	1.007	6.449	0.003	magenta
ENSG00000151322	NPAS3	neuronal PAS domain protein 3 [Source:HGNC Symbol;Acc:HGNC:19311]	protein_coding	-0.765	-0.575	0.190	0.469	3.618	0.034	magenta
ENSG00000159712	ANKRD18CP	ankyrin repeat domain 18C, pseudogene [Source:HGNC Symbol;Acc:HGNC:43601]	unprocessed_pseudogene	-1.036	-0.445	0.591	-2.379	3.475	0.038	magenta
ENSG00000196504	PRPF40A	pre-mRNA processing factor 40 homolog A [Source:HGNC Symbol;Acc:HGNC:16463]	protein_coding	0.262	0.143	-0.119	5.011	5.077	0.010	magenta
ENSG00000259001	AL355075.4	ribonuclease P RNA component H1	lncRNA	-0.425	-0.696	-0.272	3.465	3.434	0.039	magenta
ENSG00000268433	MTDHP3	metadherin pseudogene 3 [Source:HGNC Symbol;Acc:HGNC:52359]	processed_pseudogene	-1.089	0.149	1.238	-2.379	6.090	0.004	magenta
ENSG00000270757	HSPE1-MOB4	HSPE1-MOB4 readthrough [Source:HGNC Symbol;Acc:HGNC:49184]	protein_coding	0.999	2.525	1.525	-3.277	3.399	0.041	magenta
ENSG00000277209	RPPH1	ribonuclease P RNA component H1 [Source:HGNC Symbol;Acc:HGNC:19273]	ribozyme	-0.323	-0.626	-0.303	9.457	4.387	0.017	magenta
ENSG00000075213	SEMA3A	semaphorin 3A [Source:HGNC Symbol;Acc:HGNC:10723]	protein_coding	0.074	0.540	0.466	2.826	4.255	0.019	pink
ENSG00000112782	CLIC5	chloride intracellular channel 5 [Source:HGNC Symbol;Acc:HGNC:13517]	protein_coding	0.224	-0.007	-0.231	6.753	5.095	0.009	pink
ENSG00000123405	NFE2	nuclear factor, erythroid 2 [Source:HGNC Symbol;Acc:HGNC:7780]	protein_coding	0.820	1.177	0.356	-1.642	3.686	0.032	pink
ENSG00000153443	UBALD1	UBA like domain containing 1 [Source:HGNC Symbol;Acc:HGNC:29576]	protein_coding	-0.281	-0.307	-0.027	1.191	3.216	0.048	pink
ENSG00000164116	GUCY1A1	guanylate cyclase 1 soluble subunit alpha 1 [Source:HGNC Symbol;Acc:HGNC:4685]	protein_coding	0.147	0.331	0.184	5.868	3.419	0.040	pink
ENSG00000183828	NUDT14	nudix hydrolase 14 [Source:HGNC Symbol;Acc:HGNC:20141]	protein_coding	0.195	-0.163	-0.359	1.578	3.477	0.038	pink
ENSG00000196781	TLE1	TLE family member 1, transcriptional corepressor [Source:HGNC Symbol;Acc:HGNC:11837]	protein_coding	-0.240	0.093	0.334	3.139	3.421	0.040	pink

ENSG00000197249	SERPINA1	serpin family A member 1 [Source:HGNC Symbol;Acc:HGNC:8941]	protein_coding	0.291	0.769	0.477	1.284	3.742	0.030	pink
ENSG00000206337	HCP5	HLA complex P5 [Source:HGNC Symbol;Acc:HGNC:21659]	lncRNA	0.358	0.227	-0.131	2.918	4.207	0.020	pink
ENSG00000206503	HLA-A	major histocompatibility complex, class I, A [Source:HGNC Symbol;Acc:HGNC:4931]	protein_coding	0.318	0.180	-0.138	7.089	4.012	0.024	pink
ENSG00000211598	IGKV4-1	immunoglobulin kappa variable 4-1 [Source:HGNC Symbol;Acc:HGNC:5834]	IG_V_gene	-2.074	-1.365	0.709	-0.574	3.245	0.047	pink
ENSG00000211938	IGHV3-7	immunoglobulin heavy variable 3-7 [Source:HGNC Symbol;Acc:HGNC:5620]	IG_V_gene	1.385	2.422	1.037	-1.426	3.262	0.046	pink
ENSG00000254838	GVINP1	GTPase, very large interferon inducible pseudogene 1 [Source:HGNC Symbol;Acc:HGNC:25813]	transcribed_unprocessed_pseudogene	0.423	0.246	-0.177	2.093	4.444	0.016	pink
ENSG00000287129	AC097500.1	novel transcript	lncRNA	-0.719	0.140	0.859	-2.475	3.958	0.025	pink
ENSG00000100031	GGT1	gamma-glutamyltransferase 1 [Source:HGNC Symbol;Acc:HGNC:4250]	protein_coding	-0.739	-1.651	-0.912	-0.933	3.449	0.039	purple
ENSG00000112972	HMGCS1	3-hydroxy-3-methylglutaryl-CoA synthase 1 [Source:HGNC Symbol;Acc:HGNC:5007]	protein_coding	-0.034	0.411	0.445	3.535	3.296	0.045	purple
ENSG00000121858	TNFSF10	TNF superfamily member 10 [Source:HGNC Symbol;Acc:HGNC:11925]	protein_coding	0.264	-0.060	-0.324	4.139	4.796	0.012	purple
ENSG00000130164	LDLR	low density lipoprotein receptor [Source:HGNC Symbol;Acc:HGNC:6547]	protein_coding	-0.051	0.914	0.965	3.595	5.668	0.006	purple
ENSG00000165186	PTCHD1	patched domain containing 1 [Source:HGNC Symbol;Acc:HGNC:26392]	protein_coding	0.744	0.113	-0.631	-0.229	3.271	0.046	purple
ENSG00000171657	GPR82	G protein-coupled receptor 82 [Source:HGNC Symbol;Acc:HGNC:4533]	protein_coding	0.312	-0.740	-1.052	-1.881	3.665	0.032	purple
ENSG00000186480	INSIG1	insulin induced gene 1 [Source:HGNC Symbol;Acc:HGNC:6083]	protein_coding	-0.160	0.383	0.544	3.830	3.718	0.031	purple
ENSG00000213523	SRA1	steroid receptor RNA activator 1 [Source:HGNC Symbol;Acc:HGNC:11281]	protein_coding	0.014	0.254	0.240	2.751	3.229	0.047	purple
ENSG00000243364	EFNA4	ephrin A4 [Source:HGNC Symbol;Acc:HGNC:3224]	protein_coding	0.218	-0.360	-0.578	-0.823	3.544	0.036	purple
ENSG00000005801	ZNF195	zinc finger protein 195 [Source:HGNC Symbol;Acc:HGNC:12986]	protein_coding	-0.233	-0.006	0.227	2.624	3.900	0.026	red
ENSG00000009307	CSDE1	cold shock domain containing E1 [Source:HGNC Symbol;Acc:HGNC:29905]	protein_coding	-0.043	0.060	0.103	8.481	3.570	0.035	red
ENSG00000073464	CLCN4	chloride voltage-gated channel 4 [Source:HGNC Symbol;Acc:HGNC:2022]	protein_coding	0.224	0.293	0.068	4.630	4.355	0.018	red
ENSG00000077157	PPP1R12B	protein phosphatase 1 regulatory subunit 12B [Source:HGNC Symbol;Acc:HGNC:7619]	protein_coding	0.062	0.282	0.220	9.773	4.332	0.018	red
ENSG00000080166	DCT	dopachrome tautomerase [Source:HGNC Symbol;Acc:HGNC:2709]	protein_coding	-0.202	1.034	1.236	-1.950	4.340	0.018	red
ENSG00000115138	POMC	proopiomelanocortin [Source:HGNC Symbol;Acc:HGNC:9201]	protein_coding	0.250	0.960	0.710	-0.547	5.196	0.009	red

ENSG00000128482	RNF112	ring finger protein 112 [Source:HGNC Symbol;Acc:HGNC:12968]	protein_coding	0.337	-0.295	-0.633	-0.290	3.368	0.042	red
ENSG00000132274	TRIM22	tripartite motif containing 22 [Source:HGNC Symbol;Acc:HGNC:16379]	protein_coding	0.042	-0.315	-0.357	4.319	3.177	0.050	red
ENSG00000135930	EIF4E2	eukaryotic translation initiation factor 4E family member 2 [Source:HGNC Symbol;Acc:HGNC:3293]	protein_coding	-0.204	-0.371	-0.166	3.815	3.348	0.043	red
ENSG00000135931	ARMC9	armadillo repeat containing 9 [Source:HGNC Symbol;Acc:HGNC:20730]	protein_coding	-0.431	-0.804	-0.372	1.067	4.524	0.015	red
ENSG00000174437	ATP2A2	ATPase sarcoplasmic/endoplasmic reticulum Ca2+ transporting 2 [Source:HGNC Symbol;Acc:HGNC:812]	protein_coding	0.056	0.275	0.219	9.765	3.624	0.033	red
ENSG00000183508	TENT5C	terminal nucleotidyltransferase 5C [Source:HGNC Symbol;Acc:HGNC:24712]	protein_coding	0.209	0.367	0.158	4.191	5.090	0.009	red
ENSG00000189283	FHIT	fragile histidine triad diadenosine triphosphatase [Source:HGNC Symbol;Acc:HGNC:3701]	protein_coding	0.301	0.773	0.473	1.148	3.877	0.027	red
ENSG00000189419	SPATA41	spermatogenesis associated 41 [Source:HGNC Symbol;Acc:HGNC:48613]	lncRNA	0.132	-1.343	-1.475	-1.872	7.503	0.001	red
ENSG00000196196	HRCT1	histidine rich carboxyl terminus 1 [Source:HGNC Symbol;Acc:HGNC:33872]	protein_coding	-0.402	-1.096	-0.694	-1.425	3.717	0.031	red
ENSG00000213928	IRF9	interferon regulatory factor 9 [Source:HGNC Symbol;Acc:HGNC:6131]	protein_coding	0.259	-0.045	-0.304	3.190	3.321	0.044	red
ENSG00000226816	AC005082.1	novel transcript	lncRNA	-0.414	0.645	1.059	-1.461	4.575	0.015	red
ENSG00000249378	LINC01060	long intergenic non-protein coding RNA 1060 [Source:HGNC Symbol;Acc:HGNC:49081]	lncRNA	0.410	-1.306	-1.716	-3.382	3.473	0.038	red
ENSG00000277639	AC007906.2	novel protein	protein_coding	0.056	-1.114	-1.170	-1.771	3.722	0.031	red
ENSG00000286039	AC093849.2	novel transcript	lncRNA	-0.034	0.324	0.358	2.186	4.220	0.020	red
ENSG00000003402	CFLAR	CASP8 and FADD like apoptosis regulator [Source:HGNC Symbol;Acc:HGNC:1876]	protein_coding	0.191	0.220	0.029	6.684	3.404	0.041	turquoise
ENSG00000008988	RPS20	ribosomal protein S20 [Source:HGNC Symbol;Acc:HGNC:10405]	protein_coding	-0.159	-0.097	0.062	7.193	3.215	0.048	turquoise
ENSG00000047849	MAP4	microtubule associated protein 4 [Source:HGNC Symbol;Acc:HGNC:6862]	protein_coding	0.211	0.098	-0.113	8.418	3.812	0.028	turquoise
ENSG00000068781	STON1-GTF2A1L	STON1-GTF2A1L readthrough [Source:HGNC Symbol;Acc:HGNC:30651]	protein_coding	0.795	1.183	0.388	1.178	3.587	0.034	turquoise
ENSG00000076706	MCAM	melanoma cell adhesion molecule [Source:HGNC Symbol;Acc:HGNC:6934]	protein_coding	0.240	0.122	-0.118	6.150	6.109	0.004	turquoise
ENSG00000082684	SEMA5B	semaphorin 5B [Source:HGNC Symbol;Acc:HGNC:10737]	protein_coding	0.773	0.902	0.129	1.011	5.696	0.006	turquoise
ENSG00000089157	RPLP0	ribosomal protein lateral stalk subunit P0 [Source:HGNC Symbol;Acc:HGNC:10371]	protein_coding	-0.183	-0.182	0.001	7.379	3.232	0.047	turquoise
ENSG00000091622	PITPNM3	PITPNM family member 3 [Source:HGNC Symbol;Acc:HGNC:21043]	protein_coding	0.602	0.919	0.317	1.025	4.251	0.019	turquoise

ENSG00000092529	CAPN3	calpain 3 [Source:HGNC Symbol;Acc:HGNC:1480]	protein_coding	0.484	0.235	-0.249	1.996	3.428	0.040	turquoise
ENSG00000095637	SORBS1	sorbin and SH3 domain containing 1 [Source:HGNC Symbol;Acc:HGNC:14565]	protein_coding	0.173	0.075	-0.098	7.955	3.361	0.042	turquoise
ENSG00000099282	TSPAN15	tetraspanin 15 [Source:HGNC Symbol;Acc:HGNC:23298]	protein_coding	0.448	0.384	-0.064	1.459	3.883	0.027	turquoise
ENSG00000100316	RPL3	ribosomal protein L3 [Source:HGNC Symbol;Acc:HGNC:10332]	protein_coding	-0.190	-0.120	0.071	7.099	3.183	0.049	turquoise
ENSG00000100836	PABPN1	poly(A) binding protein nuclear 1 [Source:HGNC Symbol;Acc:HGNC:8565]	protein_coding	0.266	0.137	-0.129	4.163	4.963	0.011	turquoise
ENSG00000103245	CIAO3	cytosolic iron-sulfur assembly component 3 [Source:HGNC Symbol;Acc:HGNC:14179]	protein_coding	0.393	0.237	-0.156	2.397	5.280	0.008	turquoise
ENSG00000105723	GSK3A	glycogen synthase kinase 3 alpha [Source:HGNC Symbol;Acc:HGNC:4616]	protein_coding	0.228	0.216	-0.013	2.872	3.593	0.034	turquoise
ENSG00000107263	RAPGEF1	Rap guanine nucleotide exchange factor 1 [Source:HGNC Symbol;Acc:HGNC:4568]	protein_coding	0.204	0.176	-0.028	4.387	3.348	0.043	turquoise
ENSG00000107798	LIPA	lipase A, lysosomal acid type [Source:HGNC Symbol;Acc:HGNC:6617]	protein_coding	-0.296	-0.310	-0.013	4.136	3.301	0.044	turquoise
ENSG00000109099	PMP22	peripheral myelin protein 22 [Source:HGNC Symbol;Acc:HGNC:9118]	protein_coding	-0.184	-0.196	-0.012	5.509	4.483	0.016	turquoise
ENSG00000110799	VWF	von Willebrand factor [Source:HGNC Symbol;Acc:HGNC:12726]	protein_coding	0.381	0.270	-0.110	7.554	5.778	0.005	turquoise
ENSG00000112210	RAB23	RAB23, member RAS oncogene family [Source:HGNC Symbol;Acc:HGNC:14263]	protein_coding	-0.206	-0.055	0.151	3.798	3.472	0.038	turquoise
ENSG00000112306	RPS12	ribosomal protein S12 [Source:HGNC Symbol;Acc:HGNC:10385]	protein_coding	-0.175	-0.175	0.000	6.094	3.407	0.040	turquoise
ENSG00000115145	STAM2	signal transducing adaptor molecule 2 [Source:HGNC Symbol;Acc:HGNC:11358]	protein_coding	-0.126	-0.206	-0.080	4.280	3.791	0.029	turquoise
ENSG00000115904	SOS1	SOS Ras/Rac guanine nucleotide exchange factor 1 [Source:HGNC Symbol;Acc:HGNC:11187]	protein_coding	0.304	0.087	-0.217	4.676	3.315	0.044	turquoise
ENSG00000116251	RPL22	ribosomal protein L22 [Source:HGNC Symbol;Acc:HGNC:10315]	protein_coding	-0.197	-0.135	0.061	5.952	4.906	0.011	turquoise
ENSG00000118454	ANKRD13C	ankyrin repeat domain 13C [Source:HGNC Symbol;Acc:HGNC:25374]	protein_coding	-0.320	-0.208	0.112	3.447	3.928	0.026	turquoise
ENSG00000119655	NPC2	NPC intracellular cholesterol transporter 2 [Source:HGNC Symbol;Acc:HGNC:14537]	protein_coding	-0.220	-0.240	-0.020	4.374	3.235	0.047	turquoise
ENSG00000120451	SNX19	sorting nexin 19 [Source:HGNC Symbol;Acc:HGNC:21532]	protein_coding	0.243	-0.017	-0.260	5.310	5.058	0.010	turquoise
ENSG00000123427	EEF1AKMT3	EEF1A lysine methyltransferase 3 [Source:HGNC Symbol;Acc:HGNC:24936]	protein_coding	-0.248	-0.546	-0.298	0.563	3.489	0.038	turquoise
ENSG00000123636	BAZ2B	bromodomain adjacent to zinc finger domain 2B [Source:HGNC Symbol;Acc:HGNC:963]	protein_coding	0.203	0.225	0.022	5.083	3.306	0.044	turquoise
ENSG00000128591	FLNC	filamin C [Source:HGNC Symbol;Acc:HGNC:3756]	protein_coding	0.243	0.147	-0.095	7.905	3.422	0.040	turquoise

ENSG00000128891	CCDC32	coiled-coil domain containing 32 [Source:HGNC Symbol;Acc:HGNC:28295]	protein_coding	0.291	0.019	-0.272	2.514	5.944	0.005	turquoise
ENSG00000130208	APOC1	apolipoprotein C1 [Source:HGNC Symbol;Acc:HGNC:607]	protein_coding	-0.929	-0.920	0.009	-1.933	4.031	0.023	turquoise
ENSG00000130338	TULP4	TUB like protein 4 [Source:HGNC Symbol;Acc:HGNC:15530]	protein_coding	-0.135	0.019	0.154	6.330	3.307	0.044	turquoise
ENSG00000130816	DNMT1	DNA methyltransferase 1 [Source:HGNC Symbol;Acc:HGNC:2976]	protein_coding	0.540	0.392	-0.148	3.285	8.849	0.000	turquoise
ENSG00000130818	ZNF426	zinc finger protein 426 [Source:HGNC Symbol;Acc:HGNC:20725]	protein_coding	-0.227	-0.085	0.142	3.917	3.508	0.037	turquoise
ENSG00000134030	CTIF	cap binding complex dependent translation initiation factor [Source:HGNC Symbol;Acc:HGNC:23925]	protein_coding	0.303	0.197	-0.106	4.061	3.707	0.031	turquoise
ENSG00000134419	RPS15A	ribosomal protein S15a [Source:HGNC Symbol;Acc:HGNC:10389]	protein_coding	-0.199	-0.202	-0.003	6.226	3.430	0.040	turquoise
ENSG00000135837	CEP350	centrosomal protein 350 [Source:HGNC Symbol;Acc:HGNC:24238]	protein_coding	0.210	0.190	-0.021	5.693	3.326	0.043	turquoise
ENSG00000137876	RSL24D1	ribosomal L24 domain containing 1 [Source:HGNC Symbol;Acc:HGNC:18479]	protein_coding	-0.241	-0.165	0.076	4.318	3.848	0.027	turquoise
ENSG00000138363	ATIC	5-aminoimidazole-4-carboxamide ribonucleotide formyltransferase/IMP cyclohydrolase [Source:HGNC Symbol;Acc:HGNC:794]	protein_coding	0.530	0.241	-0.288	1.500	3.254	0.046	turquoise
ENSG00000138623	SEMA7A	semaphorin 7A (John Milton Hagen blood group) [Source:HGNC Symbol;Acc:HGNC:10741]	protein_coding	0.638	0.356	-0.282	-0.097	3.672	0.032	turquoise
ENSG00000138738	PRDM5	PR/SET domain 5 [Source:HGNC Symbol;Acc:HGNC:9349]	protein_coding	-0.204	0.110	0.314	3.657	3.211	0.048	turquoise
ENSG00000139714	MORN3	MORN repeat containing 3 [Source:HGNC Symbol;Acc:HGNC:29807]	protein_coding	-0.831	-0.024	0.807	-1.867	4.480	0.016	turquoise
ENSG00000140983	RHOT2	ras homolog family member T2 [Source:HGNC Symbol;Acc:HGNC:21169]	protein_coding	0.266	0.022	-0.244	3.270	3.734	0.030	turquoise
ENSG00000141668	CBLN2	cerebellin 2 precursor [Source:HGNC Symbol;Acc:HGNC:1544]	protein_coding	-1.858	-1.538	0.320	-2.318	5.888	0.005	turquoise
ENSG00000143033	MTF2	metal response element binding transcription factor 2 [Source:HGNC Symbol;Acc:HGNC:29535]	protein_coding	0.304	0.295	-0.009	2.380	3.279	0.045	turquoise
ENSG00000143162	CREG1	cellular repressor of E1A stimulated genes 1 [Source:HGNC Symbol;Acc:HGNC:2351]	protein_coding	-0.171	-0.226	-0.054	5.171	4.137	0.021	turquoise
ENSG00000143970	ASXL2	ASXL transcriptional regulator 2 [Source:HGNC Symbol;Acc:HGNC:23805]	protein_coding	0.251	0.096	-0.155	4.723	4.088	0.022	turquoise
ENSG00000146006	LRRTM2	leucine rich repeat transmembrane neuronal 2 [Source:HGNC Symbol;Acc:HGNC:19409]	protein_coding	1.167	0.938	-0.229	-1.976	4.536	0.015	turquoise
ENSG00000146278	PNRC1	proline rich nuclear receptor coactivator 1 [Source:HGNC Symbol;Acc:HGNC:17278]	protein_coding	-0.208	-0.101	0.107	6.229	3.899	0.026	turquoise
ENSG00000147419	CCDC25	coiled-coil domain containing 25 [Source:HGNC Symbol;Acc:HGNC:25591]	protein_coding	-0.215	-0.146	0.069	3.726	3.286	0.045	turquoise
ENSG00000148803	FUOM	fucose mutarotase [Source:HGNC Symbol;Acc:HGNC:24733]	protein_coding	-0.359	-0.272	0.087	0.764	3.396	0.041	turquoise

ENSG00000149532	CPSF7	cleavage and polyadenylation specific factor 7 [Source:HGNC Symbol;Acc:HGNC:30098]	protein_coding	0.220	0.102	-0.118	4.057	3.730	0.030	turquoise
ENSG00000156875	MFSD14A	major facilitator superfamily domain containing 14A [Source:HGNC Symbol;Acc:HGNC:23363]	protein_coding	-0.159	-0.014	0.145	3.701	3.246	0.047	turquoise
ENSG00000159023	EPB41	erythrocyte membrane protein band 4.1 [Source:HGNC Symbol;Acc:HGNC:3377]	protein_coding	0.312	0.283	-0.029	4.131	4.494	0.016	turquoise
ENSG00000159788	RGS12	regulator of G protein signaling 12 [Source:HGNC Symbol;Acc:HGNC:9994]	protein_coding	0.416	0.122	-0.294	2.446	5.176	0.009	turquoise
ENSG00000160007	ARHGAP35	Rho GTPase activating protein 35 [Source:HGNC Symbol;Acc:HGNC:4591]	protein_coding	0.177	0.188	0.011	6.083	3.666	0.032	turquoise
ENSG00000160321	ZNF208	zinc finger protein 208 [Source:HGNC Symbol;Acc:HGNC:12999]	protein_coding	-0.871	-0.070	0.801	2.777	8.398	0.001	turquoise
ENSG00000160408	ST6GALNAC6	ST6 N-acetylgalactosaminide alpha-2,6-sialyltransferase 6 [Source:HGNC Symbol;Acc:HGNC:23364]	protein_coding	-0.231	-0.211	0.020	4.699	4.203	0.020	turquoise
ENSG00000160803	UBQLN4	ubiquilin 4 [Source:HGNC Symbol;Acc:HGNC:1237]	protein_coding	0.209	0.014	-0.195	3.223	4.732	0.013	turquoise
ENSG00000160908	ZNF394	zinc finger protein 394 [Source:HGNC Symbol;Acc:HGNC:18832]	protein_coding	-0.197	-0.114	0.083	2.996	3.632	0.033	turquoise
ENSG00000161970	RPL26	ribosomal protein L26 [Source:HGNC Symbol;Acc:HGNC:10327]	protein_coding	-0.153	-0.141	0.013	7.280	3.221	0.048	turquoise
ENSG00000163291	PAQR3	progestin and adipoQ receptor family member 3 [Source:HGNC Symbol;Acc:HGNC:30130]	protein_coding	-0.291	-0.240	0.051	2.042	3.452	0.039	turquoise
ENSG00000163655	GMPS	guanine monophosphate synthase [Source:HGNC Symbol;Acc:HGNC:4378]	protein_coding	-0.181	-0.049	0.132	4.148	3.642	0.033	turquoise
ENSG00000164944	VIRMA	vir like m6A methyltransferase associated [Source:HGNC Symbol;Acc:HGNC:24500]	protein_coding	0.228	0.286	0.058	4.445	3.774	0.029	turquoise
ENSG00000166562	SEC11C	SEC11 homolog C, signal peptidase complex subunit [Source:HGNC Symbol;Acc:HGNC:23400]	protein_coding	-0.203	-0.029	0.174	2.184	3.284	0.045	turquoise
ENSG00000167524	RSKR	ribosomal protein S6 kinase related [Source:HGNC Symbol;Acc:HGNC:26314]	protein_coding	0.590	0.393	-0.197	-0.179	3.238	0.047	turquoise
ENSG00000169379	ARL13B	ADP ribosylation factor like GTPase 13B [Source:HGNC Symbol;Acc:HGNC:25419]	protein_coding	-0.317	-0.153	0.164	2.154	3.302	0.044	turquoise
ENSG00000170296	GABARAP	GABA type A receptor-associated protein [Source:HGNC Symbol;Acc:HGNC:4067]	protein_coding	-0.121	-0.203	-0.082	5.172	3.280	0.045	turquoise
ENSG00000170484	KRT74	keratin 74 [Source:HGNC Symbol;Acc:HGNC:28929]	protein_coding	-1.460	-0.210	1.251	-3.427	4.834	0.012	turquoise
ENSG00000171132	PRKCE	protein kinase C epsilon [Source:HGNC Symbol;Acc:HGNC:9401]	protein_coding	0.326	0.332	0.006	2.746	3.177	0.050	turquoise
ENSG00000171943	SRGAP2C	SLIT-ROBO Rho GTPase activating protein 2C [Source:HGNC Symbol;Acc:HGNC:30584]	protein_coding	-0.481	-0.436	0.044	4.035	4.918	0.011	turquoise
ENSG00000172007	RAB33B	RAB33B, member RAS oncogene family [Source:HGNC Symbol;Acc:HGNC:16075]	protein_coding	-0.167	0.021	0.188	3.208	3.567	0.035	turquoise
ENSG00000172594	SMPDL3A	sphingomyelin phosphodiesterase acid like 3A [Source:HGNC Symbol;Acc:HGNC:17389]	protein_coding	-0.379	-0.377	0.002	1.412	6.345	0.003	turquoise

ENSG00000174444	RPL4	ribosomal protein L4 [Source:HGNC Symbol;Acc:HGNC:10353]	protein_coding	-0.193	-0.152	0.041	7.818	3.688	0.032	turquoise
ENSG00000175137	SH3BP5L	SH3 binding domain protein 5 like [Source:HGNC Symbol;Acc:HGNC:29360]	protein_coding	0.204	0.161	-0.043	3.050	4.098	0.022	turquoise
ENSG00000177707	NECTIN3	nectin cell adhesion molecule 3 [Source:HGNC Symbol;Acc:HGNC:17664]	protein_coding	-0.296	-0.477	-0.181	2.999	4.526	0.015	turquoise
ENSG00000178104	PDE4DIP	phosphodiesterase 4D interacting protein [Source:HGNC Symbol;Acc:HGNC:15580]	protein_coding	0.276	0.260	-0.016	8.902	7.032	0.002	turquoise
ENSG00000179583	CIITA	class II major histocompatibility complex transactivator [Source:HGNC Symbol;Acc:HGNC:7067]	protein_coding	0.504	0.298	-0.205	2.714	3.908	0.026	turquoise
ENSG00000179776	CDH5	cadherin 5 [Source:HGNC Symbol;Acc:HGNC:1764]	protein_coding	0.308	0.277	-0.031	4.854	3.335	0.043	turquoise
ENSG00000181126	HLA-V	major histocompatibility complex, class I, V (pseudogene) [Source:HGNC Symbol;Acc:HGNC:23482]	transcribed_unprocessed_pseudogene	-1.238	-0.060	1.177	-2.874	3.899	0.026	turquoise
ENSG00000185222	TCEAL9	transcription elongation factor A like 9 [Source:HGNC Symbol;Acc:HGNC:30084]	protein_coding	-0.222	-0.171	0.051	3.550	4.252	0.019	turquoise
ENSG00000187720	THSD4	thrombospondin type 1 domain containing 4 [Source:HGNC Symbol;Acc:HGNC:25835]	protein_coding	0.313	0.215	-0.097	5.703	4.725	0.013	turquoise
ENSG00000188322	SBK1	SH3 domain binding kinase 1 [Source:HGNC Symbol;Acc:HGNC:17699]	protein_coding	1.058	0.764	-0.294	-2.553	3.464	0.038	turquoise
ENSG00000188846	RPL14	ribosomal protein L14 [Source:HGNC Symbol;Acc:HGNC:10305]	protein_coding	-0.162	-0.160	0.002	6.353	3.993	0.024	turquoise
ENSG00000189266	PNRC2	proline rich nuclear receptor coactivator 2 [Source:HGNC Symbol;Acc:HGNC:23158]	protein_coding	-0.147	-0.016	0.131	5.390	4.693	0.013	turquoise
ENSG00000197140	ADAM32	ADAM metallopeptidase domain 32 [Source:HGNC Symbol;Acc:HGNC:15479]	protein_coding	-0.573	-0.384	0.189	1.613	3.865	0.027	turquoise
ENSG00000215458	AATBC	apoptosis associated transcript in bladder cancer [Source:HGNC Symbol;Acc:HGNC:51526]	lncRNA	0.773	1.149	0.376	-2.811	3.221	0.048	turquoise
ENSG00000215912	TTC34	tetratricopeptide repeat domain 34 [Source:HGNC Symbol;Acc:HGNC:34297]	protein_coding	0.856	0.295	-0.561	-1.270	4.869	0.011	turquoise
ENSG00000225684	FAM225B	family with sequence similarity 225 member B [Source:HGNC Symbol;Acc:HGNC:21865]	lncRNA	0.183	1.725	1.542	-1.866	4.692	0.013	turquoise
ENSG00000225791	TRAM2-AS1	TRAM2 antisense RNA 1 (head to head) [Source:HGNC Symbol;Acc:HGNC:48663]	lncRNA	-0.341	-0.239	0.103	1.938	4.942	0.011	turquoise
ENSG00000229657	AL391822.1	ribosomal protein L13a (RPL13A) pseudogene	processed_pseudogene	0.992	1.247	0.255	-2.058	5.390	0.007	turquoise
ENSG00000230373	GOLGA6L5P	golgin A6 family like 5, pseudogene [Source:HGNC Symbol;Acc:HGNC:30472]	transcribed_unprocessed_pseudogene	0.104	-0.802	-0.906	-0.071	3.945	0.025	turquoise
ENSG00000241790	ENO1P4	enolase 1 pseudogene 4 [Source:HGNC Symbol;Acc:HGNC:37945]	processed_pseudogene	0.603	1.971	1.369	-4.090	4.862	0.011	turquoise
ENSG00000256977	LIMS3	LIM zinc finger domain containing 3 [Source:HGNC Symbol;Acc:HGNC:30047]	protein_coding	0.710	0.401	-0.309	0.728	3.222	0.048	turquoise
ENSG00000257379	AC023509.1	novel transcript	lncRNA	1.922	1.952	0.030	-1.213	3.360	0.042	turquoise

ENSG00000258484	SPESP1	sperm equatorial segment protein 1 [Source:HGNC Symbol;Acc:HGNC:15570]	protein_coding	-1.085	-0.084	1.001	0.451	6.878	0.002	turquoise
ENSG00000265681	RPL17	ribosomal protein L17 [Source:HGNC Symbol;Acc:HGNC:10307]	protein_coding	-0.164	-0.110	0.054	6.684	3.588	0.034	turquoise
ENSG00000266086	AC015813.2	novel transcript	protein_coding	0.796	-0.206	-1.003	0.548	5.413	0.007	turquoise
ENSG00000268555	AC123912.4	novel transcript	lncRNA	-0.088	0.509	0.597	0.314	3.445	0.039	turquoise
ENSG00000274272	AC069281.2	novel transcript	lncRNA	1.326	1.305	-0.021	-1.874	3.944	0.025	turquoise
ENSG00000280351	AC127496.7	TEC	TEC	0.843	0.990	0.147	-2.446	3.776	0.029	turquoise
ENSG00000284691	AC073111.4	novel zinc finger protein	protein_coding	0.334	-0.138	-0.471	0.670	5.207	0.009	turquoise
ENSG00000287839	AL353807.5	novel transcript	lncRNA	0.661	0.185	-0.477	-1.399	3.480	0.038	turquoise
ENSG00000057294	PKP2	plakophilin 2 [Source:HGNC Symbol;Acc:HGNC:9024]	protein_coding	0.256	0.132	-0.124	7.028	3.897	0.026	yellow
ENSG00000073910	FRY	FRY microtubule binding protein [Source:HGNC Symbol;Acc:HGNC:20367]	protein_coding	0.190	0.312	0.122	6.255	5.019	0.010	yellow
ENSG00000101608	MYL12A	myosin light chain 12A [Source:HGNC Symbol;Acc:HGNC:16701]	protein_coding	0.454	0.080	-0.374	7.847	5.757	0.005	yellow
ENSG00000101871	MID1	midline 1 [Source:HGNC Symbol;Acc:HGNC:7095]	protein_coding	-0.291	-0.147	0.144	4.153	3.352	0.042	yellow
ENSG00000103710	RASL12	RAS like family 12 [Source:HGNC Symbol;Acc:HGNC:30289]	protein_coding	0.258	0.124	-0.134	4.180	3.637	0.033	yellow
ENSG00000107165	TYRP1	tyrosinase related protein 1 [Source:HGNC Symbol;Acc:HGNC:12450]	protein_coding	0.361	0.041	-0.321	3.461	3.754	0.030	yellow
ENSG00000116977	LGALS8	galectin 8 [Source:HGNC Symbol;Acc:HGNC:6569]	protein_coding	0.232	0.058	-0.174	5.499	4.494	0.016	yellow
ENSG00000123901	GPR83	G protein-coupled receptor 83 [Source:HGNC Symbol;Acc:HGNC:4523]	protein_coding	0.585	-0.050	-0.635	-0.119	3.548	0.036	yellow
ENSG00000133169	BEX1	brain expressed X-linked 1 [Source:HGNC Symbol;Acc:HGNC:1036]	protein_coding	-0.842	-0.386	0.456	1.433	3.878	0.027	yellow
ENSG00000134571	MYBPC3	myosin binding protein C3 [Source:HGNC Symbol;Acc:HGNC:7551]	protein_coding	0.233	0.156	-0.077	8.845	3.908	0.026	yellow
ENSG00000136040	PLXNC1	plexin C1 [Source:HGNC Symbol;Acc:HGNC:9106]	protein_coding	-0.339	0.023	0.362	2.522	3.345	0.043	yellow
ENSG00000136144	RCBTB1	RCC1 and BTB domain containing protein 1 [Source:HGNC Symbol;Acc:HGNC:18243]	protein_coding	0.222	0.135	-0.087	3.780	4.322	0.018	yellow
ENSG00000136932	TRMO	tRNA methyltransferase O [Source:HGNC Symbol;Acc:HGNC:30967]	protein_coding	-0.243	-0.202	0.041	1.848	3.464	0.038	yellow
ENSG00000141664	ZCCHC2	zinc finger CCHC-type containing 2 [Source:HGNC Symbol;Acc:HGNC:22916]	protein_coding	0.170	-0.115	-0.284	3.247	3.764	0.030	yellow

ENSG00000146197	SCUBE3	signal peptide, CUB domain and EGF like domain containing 3 [Source:HGNC Symbol;Acc:HGNC:13655]	protein_coding	0.685	0.080	-0.605	0.040	3.592	0.034	yellow
ENSG00000148660	CAMK2G	calcium/calmodulin dependent protein kinase II gamma [Source:HGNC Symbol;Acc:HGNC:1463]	protein_coding	0.295	0.242	-0.053	2.768	4.071	0.023	yellow
ENSG00000148925	BTBD10	BTB domain containing 10 [Source:HGNC Symbol;Acc:HGNC:21445]	protein_coding	-0.219	0.021	0.240	3.754	5.681	0.006	yellow
ENSG00000163145	C1QTNF7	C1q and TNF related 7 [Source:HGNC Symbol;Acc:HGNC:14342]	protein_coding	-0.494	-0.335	0.159	2.377	4.599	0.014	yellow
ENSG00000163681	SLMAP	sarcolemma associated protein [Source:HGNC Symbol;Acc:HGNC:16643]	protein_coding	0.367	0.186	-0.181	7.039	3.787	0.029	yellow
ENSG00000166974	MAPRE2	microtubule associated protein RP/EB family member 2 [Source:HGNC Symbol;Acc:HGNC:6891]	protein_coding	0.200	0.076	-0.124	6.062	3.448	0.039	yellow
ENSG00000175084	DES	desmin [Source:HGNC Symbol;Acc:HGNC:2770]	protein_coding	0.238	0.191	-0.047	10.761	7.549	0.001	yellow
ENSG00000175182	FAM131A	family with sequence similarity 131 member A [Source:HGNC Symbol;Acc:HGNC:28308]	protein_coding	0.271	0.294	0.022	2.613	3.290	0.045	yellow
ENSG00000196109	ZNF676	zinc finger protein 676 [Source:HGNC Symbol;Acc:HGNC:20429]	protein_coding	-0.418	-0.103	0.315	1.288	3.306	0.044	yellow
ENSG00000197977	ELOVL2	ELOVL fatty acid elongase 2 [Source:HGNC Symbol;Acc:HGNC:14416]	protein_coding	0.501	0.026	-0.475	0.202	3.724	0.031	yellow
ENSG00000198467	TPM2	tropomyosin 2 [Source:HGNC Symbol;Acc:HGNC:12011]	protein_coding	0.213	0.130	-0.082	7.110	3.314	0.044	yellow
ENSG00000198952	SMG5	SMG5 nonsense mediated mRNA decay factor [Source:HGNC Symbol;Acc:HGNC:24644]	protein_coding	0.207	0.103	-0.104	5.130	4.797	0.012	yellow
ENSG00000205084	TMEM231	transmembrane protein 231 [Source:HGNC Symbol;Acc:HGNC:37234]	protein_coding	-0.394	-0.108	0.286	2.287	4.462	0.016	yellow
ENSG00000244306	AL589743.1	double homeobox A pseudogene 10	transcribed_processed_pseudogene	-1.053	-0.920	0.133	0.051	4.192	0.020	yellow
ENSG00000260596	DUX4	double homeobox 4 [Source:HGNC Symbol;Acc:HGNC:50800]	protein_coding	-1.036	0.593	1.629	-2.604	3.444	0.039	yellow
ENSG00000261485	PAN3-AS1	PAN3 antisense RNA 1 [Source:HGNC Symbol;Acc:HGNC:39932]	lncRNA	0.771	0.511	-0.260	-1.666	5.096	0.009	yellow
ENSG00000285238	AC006064.6	novel transcript	protein_coding	1.987	2.646	0.659	-2.431	4.253	0.019	yellow
